# CRISPR-Cas Systems Features and the Gene-Reservoir Role of Coagulase-Negative Staphylococci

**DOI:** 10.3389/fmicb.2017.01545

**Published:** 2017-08-15

**Authors:** Ciro C. Rossi, Thaysa Souza-Silva, Amanda V. Araújo-Alves, Marcia Giambiagi-deMarval

**Affiliations:** Instituto de Microbiologia Paulo de Góes, Universidade Federal do Rio de Janeiro Rio de Janeiro, Brazil

**Keywords:** CRISPR, *cas* genes, CoNS, coagulase-negative staphylococci, horizontal gene transfer

## Abstract

The claimed role of gene reservoir of coagulase-negative staphylococci (CoNS) could be contradicted by estimates that CRISPR/Cas systems are found in the genomes of 40–50% of bacteria, as these systems interfere with plasmid uptake in staphylococci. To further correlate this role with presence of CRISPR, we analyzed, by computational methods, 122 genomes from 15 species of CoNS. Only 15% of them harbored CRISPR/Cas systems, and this proportion was much lower for *S. epidermidis* and *S. haemolyticus*, the CoNS most frequently associated with opportunistic infections in humans. These systems are of type II or III, and at least two of them are located within SCC*mec*, a mobile genetic element of *Staphylococcus* bacterial species. An analysis of the spacers of these CRISPRs, which come from exogenous origin, allowed us to track the transference of the SCC*mec*, which was exchanged between different strains, species and hosts. Some of the spacers are derived from plasmids described in *Staphylococcus* species that are different from those in which the CRISPR are found, evidencing the attempt (and failure) of plasmid transference between them. Based on the polymorphisms of the *cas1* gene in CRISPRs of types II and III, we developed a multiplex polymerase chain reaction (PCR) suitable to screen and type CRISPR systems in CoNS. The PCR was tested in 59 *S. haemolyticus* strains, of which only two contained a type III *cas1*. This gene was shown to be expressed in the exponential growth, stationary phase and during biofilm formation. The low abundance of CRISPRs in CoNS is in accordance with their role as gene reservoirs, but when present, their spacers sequence evidence and give an insight on the dynamics of horizontal genetic transfer among staphylococci.

## Introduction

Coagulase-negative staphylococci (CoNS) were for a long time considered as harmless residents of the normal microbiota of skin and mucous membranes of humans, but are increasingly being recognized as central causative agents of healthcare-associated infections (Becker et al., 2014; Asaad et al., 2016). Among them, *Staphylococcus epidermidis* and *Staphylococcus haemolyticus* have become two major causes of nosocomial infections, which are difficult to eradicate given their ability to acquire multiresistance against available antimicrobial agents and capacity to form biofilms on indwelling medical devices (Vuong and Otto, 2002; Czekaj et al., 2015).

The establishment of biofilms also provide an environment of high cell density, increased genetic competence and availability of mobile genetic elements that altogether make an ideal setting for horizontal transference of resistance genes (Flemming et al., 2016). In fact, evidences show that CoNS may act as reservoirs of genes that may be transferred between related and to more pathogenic bacteria, such as *Staphylococcus aureus*, thus enhancing its potential to resist antimicrobial treatment (Otto, 2013; Rossi et al., 2016a). The evidences for transfer of important elements, such as plasmids and the staphylococcal cassette chromosome *mec* (SCC*mec*, which harbors the *mecA* gene coding for an alternative penicillin binding protein), from CoNS to *S. aureus* are quite strong as observed by sequence homology (Barbier et al., 2010; Otto, 2013).

Conversely, horizontal gene transfer is greatly limited in staphylococci by the presence of clustered regularly interspaced short palindromic repeats – CRISPR (Marraffini and Sontheimer, 2008; Cao et al., 2016), which are believed to be present in approximately 40–50% and 90% of bacterial and archaeal genomes, respectively (Sorek et al., 2008).

As suggested by its name, CRISPR is an array of short (24–47 bp) direct repeats (DRs), which are separated from each other by equally short segments of DNA derived from previous exposure to foreign DNA, specially a virus or plasmid (van der Oost et al., 2014). Functional CRISPR systems are always closely accompanied by a cluster of CRISPR-associated proteins (Cas), each in charge of different functions for the system. The complex formed by the Cas1 and Cas2 proteins recognizes fragments of an invader genetic material, previously processed by proteins of the cell repair system. These fragments are incorporated as “spacers” in the bacterial/archaeal genome, within the CRISPR array (Amitai and Sorek, 2016), which is then transcribed as a large transcript containing several spacers separated by palindromic DRs. The transcript is processed into small CRISPR RNAs (crRNA) containing a spacer and a hairpin structure, used for interference in a subsequent invasion of a mobile genetic element containing the same or a sequence similar to the one in the crRNA (Barrangou and Horvath, 2017).

Based on the Cas proteins involved in each step of CRISPR adaptation, expression, maturation and interference, two classes and six types of CRISPR systems are currently recognized. While in class I (comprising types I, III, and IV) systems targeting is performed by multiprotein complexes, in class II (types II, V, and VI) this role is played by a single protein (Barrangou and Horvath, 2017). Interference by type II CRISPR is performed by Cas9 and associated RNAs. Because of its simplicity, this system has been vastly adapted to be used as a tool for mostly modifying sequences and/or controlling expression of target genes (Luo et al., 2016). However, some particularities of Cas9 of different sources, such as the need of recognizing specific sequences – the protoadjacent motif, PAM – and the difficulties in applying this modification system in prokaryotes, are boosting the use of Cas proteins of other types and the search for new systems (Luo et al., 2016; Burstein et al., 2017; Cloney, 2017).

To further understand the importance of coagulase negative staphylococci as gene reservoirs and the relationship between this role and the presence of CRISPR within the bacterial genome, this work aimed to identify and characterize CRISPR systems in several CoNS of species that have been described in humans, as well as establishing an efficient detection method, evaluating its abundance and expression.

## Materials and Methods

### CoNS Genomes Analyzed

A total of 122 sequenced genomes available from GenBank (Supplementary Table S1) were used for the computational analysis. They belong to 15 different species of coagulase negative *Staphylococcus* that have been detected in humans, among other sources (Becker et al., 2014): *S. auricularis* (*n* = 1), *S. capitis* (*n* = 7), *S. epidermidis* (*n* = 34), *S. haemolyticus* (*n* = 32), *S. hominis* (*n* = 6), *S. lugdunensis* (*n* = 9), *S. massiliensis* (*n* = 1), *S. pettenkoferi* (*n* = 1), *S. saprophyticus* (*n* = 8), *S. schleiferi* (*n* = 5), *S. sciuri* (*n* = 1), *S. simulans* (*n* = 3), *S. succinus* (*n* = 1), *S. warneri* (*n* = 10), and *S. xylosus* (*n* = 3).

### CRISPR Search and Characterization

All the 122 CoNS genomes were analyzed with the CRISPRFinder tool (Grissa et al., 2007) for the search of possible CRISPR structures. Then, the loci of those genomes that were positive for CRISPR candidates were collected and further annotated with Artemis (Rutherford et al., 2000) for the search of *cas* genes, and with ISFinder (Siguier et al., 2012) for the search of insertion sequences surrounding the system.

### Direct Repeats and Spacer Analysis

The DRs of every CRISPR found were aligned using Clustal Omega (Sievers and Higgins, 2014); then a phylogenetic inference was done using Mr. Bayes 3.2 (Ronquist et al., 2012). The alignment was also used to build sequence logos with Weblogo 3.0 (Crooks et al., 2004). Secondary structure of each DR was predicted by RNAFold (Gruber et al., 2008). Additionally, the origin of every spacer sequence was investigated by nucleotide BLAST against the NCBI’s database.

### CRISPR Screening in Clinical Strains

The sequences of the *cas1* gene from all the CRISPR-positive genomes were aligned by Clustal Omega and the best conserved regions were analyzed to design the oligonucleotide pairs cas1IIF (5′-AAT ATA GAG GGC CAA GCG GC-3′)/cas1IIR (5′-CGC ATG CAG CAA GTT AAT CAG C-3′) to detect a 324 bp fragment of the *cas1* gene from type II CRISPR systems, and cas1IIIF (5′-TGT TAC TGC GAA GGA AAA TAG C-3′)/cas1IIIR (5′-CGT CCA CGT TTA AAT TGT TTG CC-3′), to detect a 471 bp *cas1* gene of type III CRISPR systems. Both cas1IIF/R and cas1IIIF/R primer pairs were used in a multiplex polymerase chain reaction (PCR) reaction containing 2 U of the GoTaq G2 DNA Polymerase (Promega, United States) in the enzyme buffer, containing 0.2 mM of each dNTP, 0.2 mM of each primer and 0.1 mM of the primer pair 16S rRNAF/R (Chen et al., 2013) as an endogenous control. Screening was performed in 59 clinical strains of *Staphylococcus haemolyticus* previously analyzed by our group and shown to be phenotypic and genetically diverse (Barros et al., 2012; Rossi et al., 2016b). Amplification was performed in a T100 Thermal Cycler (BioRad, United States) with the following cycle parameters: initial denaturation at 95°C for 3 min, followed by 30 cycles (95°C for 30 s, 50°C for 1 min, and 72°C for 1 min) and 72°C for 3 min. The reaction products were analyzed after electrophoresis in 1.0% agarose gel. *Staphylococcus epidermidis* RP62A (Gill et al., 2005) was used as a positive control for type III-CRISPR *cas1* and *S. epidermidis* 10 L (Rossi et al., 2017); as a positive control for type II-CRISPR *cas1*.

### Real Time Polymerase Chain Reaction

The expression of *cas1* was evaluated in triplicate by RT-PCR in *Staphylococcus haemolyticus* clinical strains positive for the gene. Overnight cultures grown on Brain Heart Infusion (BHI-BD, France) agar plates were used to prepare bacterial suspensions using a 0.5 McFarland turbidity standard. The bacterial suspensions were then diluted in 1:100 fresh BHI and incubated at 37°C under agitation (150 rpm) for 4 h (exponential growth), 16 h (stationary phase) and 48 h for the formation of biofilms in the walls of the glass flasks used for cultivation. Cells were then harvested and ressuspended in RNAlater (Thermo Fisher Scientific, United States) and kept at -20°C until RNA extraction. RNA extraction was performed with the RNAeasy mini kit (Qiagen, Germany) according to the manufacturer’s instructions, with cells being previously disrupted with Lysing Matrix Tubes B (MP Biomedicals, United States) in a Mini-beadbeater-24 (BioSpec, United States) in three rounds of 40 s. RNA quantity and quality were evaluated by spectrophotometry and gel electrophoresis, respectively. Then, RNA was treated with RQ1 RNAse-Free DNAse (Promega, United States), following the manufacturer’s instructions. Complete removal of DNA was evaluated by PCR with primers for the *rpoB* gene, rpoB-2643F and rpoB-3241R, with amplification parameters as suggested by the author of the primers (Drancourt and Raoult, 2002). cDNA first strand synthesis was performed with the DNAse-treated RNA, using the ImProm-II^TM^ Reverse Transcription System (Promega, United States). Then, a PCR reaction was conducted with the primer pair cas1IIIF/cas1IIIR, 1 U of GoTaq G2 DNA Polymerase (Promega, United States) in the enzyme buffer, containing 0.2 mM of each dNTP and 0.2 mM of each primer. Amplification was done as described above. The reaction products were analyzed after electrophoresis in 1.0% agarose gel. As negative controls, we used RNA that was subjected to the same reactions of cDNA synthesis, but without the addition of the Reverse Transcriptase.

## Results

### CRISPR/Cas Systems in CoNS

**FIGURE 1:**
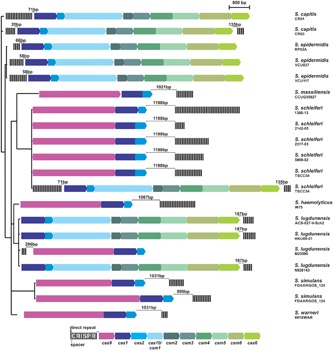
CRISPR/Cas systems in coagulase-negative staphylococci. Black and gray strips represent the direct repeats (DRs) and spacers of each CRISPR (respectively) and arrows represent the genes for each Cas protein. For comparison purposes, strains CRISPRs were organized in a three built from the Bayesian inference of the phylogeny of each strain 16S rRNA sequence.

A total of 122 public available genomes, belonging to 15 strains of coagulase negative staphylococci species that have already been described in the human microbiota (among other sources), were analyzed for the presence of CRISPR-like sequences by the CRISPRfinder software. Among them, 32 strains were suspicious of containing CRISPR/Cas systems (Supplementary Table S1). However, many of these sequences were too small (comprising three DRs and two spacers), and most of them are not adjacent to *cas* genes. Therefore, these repetitive sequences are not included in real CRISPR/Cas systems. Overall, only 18 (15%) strains, belonging to eight species (*S. capitis*, *S. epidermidis*, *S. massiliensis*, *S. schleiferi*, *S. haemolyticus*, *S. lugdunensis*, *S. simulans*, and *S. warneri*) contain CRISPR/Cas systems, characterized by 3 (*S. lugdunensis* M23590) to 37 (*S. schleiferi* 1360-13) DRs and an adjacent cluster of *cas* genes (**Figure 1** and Supplementary Figure S1). The proportion is even lower in CoNS that act as important human pathogens: among the *S. epidermidis* strains, only 9% (3/34) of them presented characteristic CRISPR/Cas systems, and among *S. haemolyticus*; only 3% (1/32).

These systems are all complete and are either of type II, containing the *cas1, cas2*, and *cas9* genes; or of type III, which also contains the universal *cas1-2* genes, in addition to *cas6*, *cas10*, and *csm2-6*. Curiously, some systems (the type III ones from *S. capitis* CR03 and *S. schleiferi* TSCC54) contain repetitive sequences both up and downstream the *cas-*gene cluster, although one of them seems to be more active than the other, given the difference in the number of spacers presented. In addition, two strains contain two independent CRISPR/Cas systems in their genome: while the *S. simulans* FDAARGOS_124 strain contains two type II systems, the *S. schleiferi* TSCC54 strain contains one type II system (like all other strains from this species), but harbors an additional system, of type III.

### CRISPR/Cas Systems Exchange between Species

**FIGURE 2:**
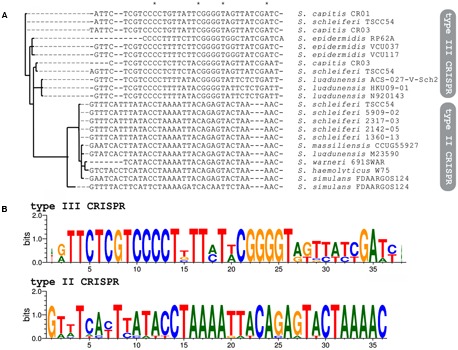
Sequence diversity of the DRs of coagulase-negative staphylococci CRISPRs. **(A)** Bayesian inference of the phylogeny of each strain DR sequence. Asterisks indicate identical bases in the sequence alignment. **(B)** Sequence logo of DR of type II and type III CRISPR systems.

The presence of a CRISPR/Cas system in *S. schleiferi* TSCC54 different from the one that is common for the species (**Figure 1**) lead us to investigate whether they could have had different origins. For that purpose, we first analyzed the DRs of each CRISPR found and made a Bayesian inference of the phylogeny of those sequences, which are usually conserved among species. Most DR formed groups in a species-dependent manner, in addition to defining two clades: one of type II CRISPR system DR, and other from type III (**Figure 2A**).

All the DR from type III systems have very conserved regions containing the motif CCCC, separated by eight nucleotides from a GGGG stretch. Both sequences are likely to interact with one another, to form the typical hairpin structures that are both important for the initial processing of the CRISPR transcript and the interaction of the CRISPR small RNA (crRNA, involved in the interference process) with the Cas proteins. Although the same motifs are not observed in the DR from type II systems, which are more varied, all the DR are also capable of forming the typical secondary structures (**Figure 2B** and Supplementary Figure S2).

**FIGURE 3:**
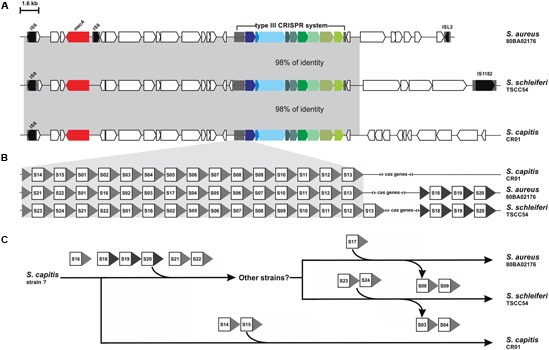
CRISPR systems within SCC*mec* elements. **(A)** Comparison of the genetic context of the CRISPR-containing *SCCmec* of *S. schleiferi* TSCC54 and *S. capitis* CR01 analyzed in this work and *S. aureus* 80BA02176. The *mecA* gene is highlighted in red. **(B)** Analysis of the spacer sequences – squares – present in each CRISPR show that many of them are identical. **(C)** Hypothesis of the history of SCC*mec*-mediated CRISPR/Cas system transference between *S. capitis* CR01, *S. aureus* 80BA02176 and *S. schleiferi* TSCC54 and the acquisition of new spacers.

The DR from *S. schleiferi* TSCC54 type III system grouped with the DR from *S. capitis* CR03, which raised the hypothesis of a horizontal transference between them. To investigate the location of CRISPR loci within mobile genetic elements, we first searched for insertion sequences in the vicinity of these elements. Many of the systems found in here are nearby insertion sequences (Supplementary Table S2), but an in depth analysis and annotation of genes around the CRISPR/Cas systems of *S. schleiferi* TSCC54 and *S. capitis* CR03 showed that they both are located within SCC*mec* elements (**Figure 3A**). These elements display an almost identical region (98% of identity) that is also very similar to a SCC*mec* previously described for the *S. aureus* strain 80BA02176 (Golding et al., 2012). Surprisingly, these three systems share most of their spacer sequences, evidencing a common origin (**Figure 3B**). Given the number and array of spacers, we hypothesize that this CRISPR/Cas element was originally from a *S. capitis* strain that diverged into two different strains (**Figure 3C**): (i) one that acquired the spacers S14 and S15, originating the *S. capitis* CR01 strain and (ii) another that acquired the spacers S16 and S18-S22, originating the strain that served as the donor of the CRISPR-containing SSC*mec* to *S. aureus* 80BA02176 (which incorporated the S17 spacer and lost S08-S09) and to *S. schleiferi* TSCC54 (which acquired S23-24 and lost S03-S04). It is also possible that other intermediate strains were involved in this process of SSC*mec* and CRISPR/Cas systems exchange process.

### The Origin of CoNS Spacers

Considering that some spacers are identical between the *S. capitis* CR01 and *S. schleiferi* TSCC54 strains and that spacers were found repeatedly within the same species (among *S. schleiferi* strains) and even in the same CRISPR of a single strain (*S. massiliensis* CCUG55927), there are overall 194 unique spacer sequences. Among them, the great majority (93%) are from an unknown origin, as they do not present homology with any sequence available from GenBank. Only 13 (7%) sequences are known, of which seven (4%) are identical to a variety of plasmids found in different species belonging to the *Staphylococcus* genus (Supplementary Table S3). A spacer from *S. haemolyticus* W75 (shae20), for example, has a sequence that is identical to that of the *S. aureus* plasmid pSAM12-0145 (GenBank ID KU521355.1) and the *S. cohnii* plasmid pHK01 (GenBank ID KC820816.1). The remaining six (3%) spacers originally may have come from bacteriophages (Supplementary Table S3), some of which were isolated from other *Staphylococcus* species.

### A Multiplex PCR for Screening and Typing CRISPR Systems in CoNS

**FIGURE 4:**
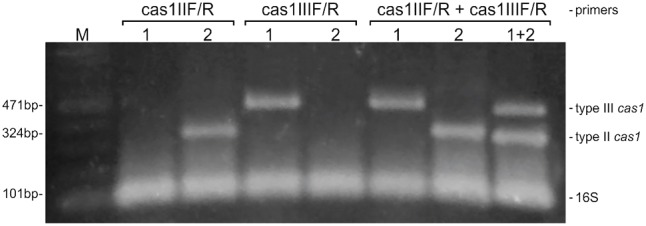
A multiplex PCR for screening and typing CRISPR/Cas systems in coagulase-negative staphylococci. The multiplex was designed to detect the universal *cas1* gene, and to differentiate than between those belonging to types II and III CRISPR systems. The detection of the 16S rRNA is employed as an endogenous control of the reaction. *S. epidermidis* strains RP62A (1) and 10 L (2) were used as positive controls for the amplification of *cas1* of types II and III, respectively. M: molecular weight marker.

As well as having very distinct DR sequences, CRISPR systems from CoNS also present sequences for the *cas1* gene that differ from on type to the other. A comparison between the *cas1* gene sequences from all the strains analyzed in this work showed that when the sequences from both type II and type III *cas1* are aligned together, they present an identity as low as 29% (data not shown). However, when the sequences are analyzed in their own separate CRISPR-type groups, this value increases to 54% for type II *cas1*, and to 70% for type III. These differences lead us to design primers to *cas1* of each group separately, so that the amplification of that gene could be performed in a multiplex PCR reaction that allowed us not only to detect more efficiently strains harboring that CRISPR marker gene, but also permitted their differentiation according to the type of system (**Figure 4**). In addition, the reaction allows the identification of strains containing more than one type of CRISPR system in its genome, as in the case observed computationally for the *S. schleiferi* TSCC54 strain, described above.

Because *S. haemolyticus* is one of the most important CoNS and no CRISPRs have been described to the species so far, the multiplex PCR reaction was employed in 59 clinical strains of this species previously shown by our group to be phenotypic and genetically diverse (Rossi et al., 2016b). The *cas1* gene was amplified in only 3% of the strains (2/59), namely MD03 and MD25. The proportion found is the same observed in the *in silico* analysis for the species. However, while in the genome available for *S. haemolyticus* W75 the CRISPR system belongs to the type II, in both of our clinical strains the amplicon size indicates they belong to type III (data not shown).

### The *cas1* Gene Is Expressed during All Phases of *S. haemolyticus* Growth

**FIGURE 5:**
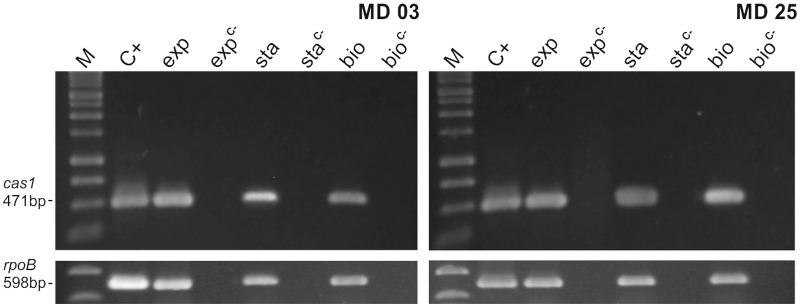
Expression of *cas1* in *S. haemolyticus* clinical strains MD03 and MD25. The expression of type III *cas1* in the clinical strains that were positive for the multiplex PCR reaction was evaluated during exponential growth (exp), stationary phase (sta) and biofilm formation (bio). M: molecular weight marker, C+: positive control (genomic DNA) and C–: negative control (RNA that was subjected to the cDNA synthesis reaction, but without the addition of the Reverse Transcriptase). The expression of the *rpoB* gene was used as and endogenous control.

To get an insight on the functionality of the CRISPR systems found in the screening, we evaluated the expression of the *cas1* gene in strains MD03 and MD05 during exponential growth, stationary phase and in the biofilm form. As expected for a surveillance-related gene, the expression of *cas1* was observed in all situations (**Figure 5**).

## Discussion

Coagulase-negative staphylococci are advocated to act as resistance and virulence gene reservoirs, but this role could be contradicted by estimates that CRISPR/Cas systems are present in the genomes of more than 40% of bacteria (Sorek et al., 2008), since these systems greatly interfere with plasmid uptake in staphylococci (Marraffini and Sontheimer, 2008; Cao et al., 2016). However, recent studies indicate that this abundance may be considerably lower in major bacterial lineages that have no cultivated representatives (Burstein et al., 2016). Also consistent with the evidences of constant horizontal gene transfer between staphylococci, a recent study with 636 *Staphylococcus aureus* strains isolated from hospitals in China found by PCR only 6 (close to 1%) strains with a complete arrange of *cas* genes (Cao et al., 2016).

CRISPR abundance could also be overestimated, as a number of very small “questionable” CRISPRs can be detected computationally, but need to be critically investigated for the presence of *cas* genes. Here we found that 15% of the strains investigated contain real CRISPRs, but we believe that this number could still be overestimated among CoNS, as most of the CRISPR-containing *S. schleiferi* strains, 1360-13, 2142-05, 2317-03, and 5909-02 were isolated by the same group in the United States (Misic et al., 2015). The largest and more varied group of sequences available are from *S. epidermidis* and *S. haemolyticus*, and both showed to have a considerably lower proportion of CRISPR systems. These two are the most significant species of CoNS and account substantially for foreign body-related infections and infections in preterm newborns (Becker et al., 2014). They also represent a therapeutic challenge due to the large proportion of methicillin-resistant strains and the great number of antimicrobial resistance genes that they usually harbor (Becker et al., 2014; Czekaj et al., 2015).

Six types of CRISPR systems are virtually accepted (Luo et al., 2016), although most of the bacteria and archaea contain either systems of types I, II, or III, with type II being mostly found in bacteria (Makarova et al., 2015). A metagenomic study has recently revealed the existence of two novel CRISPR-Cas systems containing new Cas proteins, named as CasX and CasY (Cloney, 2017). But like types IV and V systems, their mechanism of action still have to be elucidated. Here we show that CoNS contain either CRISPR-Cas systems of type II (containing Cas1-2 and its signature Cas9 protein), or type III (containing the genes for the proteins Cas1-2, Cas10, Cas6, and Csm2-6). The fact that many of them are surrounded by insertion sequences indicates that they can potentially be transferred horizontally and be eventually lost in the absence of a selective pressure. However, because most of the genomes analyzed are available in the form of draft sequences, deeper analysis of the surroundings of these CRISPRs is not possible.

On the other hand, it is undeniable the existence of CRISPRs within SCC*mec* elements in two strains studied (*S. capitis* CR01 and *S. schleiferi* TSCC54). Even more remarkable is the fact that the sequence of these strains spacers allowed us to observe that these systems have a common origin and that they were transferred between strains and species. This is not only one more evidence of horizontal gene transfer between CoNS and *S. aureus*, but also between human and animal strains, as *S. schleiferi* TSCC54 was isolated from the skin of a dog in Japan. Even though the species can be recovered from both humans and dogs, it is only clinically significant to the latter, for causing various disease states such as pyoderma, otitis, and urinary tract infections (Sasaki et al., 2015).

Then, the diversity of spacers, as described above, allows tracking the history of genetic material between different strains, species and hosts. Meanwhile, for the bacteria, the more diverse their spacers are, the more efficient is the defense of the population against mobile genetic elements (Lopatina and Sorek, 2016). Most spacers from CoNS are from an unknown origin, which once more reflects the general under-sampling of phage and plasmid sequences and in consequence, their availability in public databases (Mojica et al., 2005). In addition, the fact that some strains contain spacers derived from plasmids found in different species evidences that those CRISPR-containing cells indeed restrict horizontal gene transfer between staphylococci, in accordance to experimental evidences (Marraffini and Sontheimer, 2008; Cao et al., 2016).

Screening for CRISPR-positive strains will help understanding the importance of genetic exchange within that population and, when they are present, it will also help tracking the dynamics of that transfer, by analyzing the sequence of the spacers. Moreover, identifying these systems in species for which no CRISPR elements have been described could increase the repertoire of natural Cas proteins that recognize different PAMs. Choosing the more suitable Cas to perform molecular edition will hopefully facilitate the manipulation of genomes for species to which the systems available are not efficient, especially in prokaryotes (Luo et al., 2016).

The *cas1* gene is ideal for screening purposes for being the only one that is virtually in every CRISPR/Cas system described so far. The *cas2* gene is also widespread, but it is not present in one of the recently described systems (Cloney, 2017). The multiplex PCR developed in here overcomes the sequence polymorphisms observed for the *cas1* gene among CoNS, being a suitable strategy for identifying and typing CRISPRs among this group of bacteria. Because *S. haemolyticus* is an important reservoir of several antimicrobial resistance genes that can be transferred to *S. aureus* (Czekaj et al., 2015; Rossi et al., 2016a), we expected that clinical strains of this species would mostly lack CRISPR systems in their genomes. Indeed, our search for the *cas1* gene resulted in a proportion of positive strains as little as that observed by bioinformatics. We also observed that the expression of *cas1* takes place in all growth phases of *S. haemolyticus*, as it would be expected for a system responsible for surveilling the presence of genetic invaders. However, in some bacteria, like *E. coli*, envelope stress is a trigger for CRISPR expression (Perez-Rodriguez et al., 2011); and in *Myxococcus xanthus* the *cas* genes are located in a larger operon, that is also responds to stressful conditions (Viswanathan et al., 2007). Future studies will elucidate whether biofilm formation, an important virulence determinant of *S. haemolyticus*, influences the expression of the CRISPR and its associated genes.

## Conclusion

This work shows that the abundance of CRISPR/Cas systems in CoNS is considerably lower than the average expected for bacteria in general, which is in accordance with their role as gene reservoirs. However, when present, the sequence of the CRISPR spacers evidence and give an insight on the dynamics of horizontal genetic transfer among staphylococci, which undeniably happens between strains, species, and hosts.

## Author Contributions

CR conceived the study, designed and performed experiments, purchased materials, and wrote the manuscript. TS-S designed and tested the multiplex PCR reactions. AA-A helped with conceiving of the study and manuscript draft. MG-dM conceived the study, purchased materials, and participated in the study’s design and coordination. All authors read and approved the final manuscript.

## Conflict of Interest Statement

The authors declare that the research was conducted in the absence of any commercial or financial relationships that could be construed as a potential conflict of interest. The reviewer PM-A and handling Editor declared their shared affiliation, and the handling Editor states that the process nevertheless met the standards of a fair and objective review.
