# Supplementary material for: CRISPR-Cas Systems Features and the Gene-Reservoir Role of Coagulase-Negative Staphylococci
Source: Front Microbiol. 2017 Aug 15;8:1545. doi: 10.3389/fmicb.2017.01545 (PMC5559504; doi:10.3389/fmicb.2017.01545)
Supplement: Supplementary file 1 [file Table_1.PDF]

Table S1. Coagulase-negative *Staphylococcus* genomes from strains of human origin analyzed in this work.

Species	Strains (access)	CRISPR-like arrangement (nº repeats/nº spacers)	*cas* genes
S. auricularis	DSM206609 (NZ_LLER01000002)	-	-
S. capitis	AYP1020 (CP007601)	(3/2); (3/2)	-
	C87 (NZ_ACRH00000000)	(6/5)	-
	CR01 (NZ_CBUB000000000)	(16/15); (4/3); (3/2)	**+**
	CR03 (NZ_CVUF00000000)	(8/7); (6/5); (4/3); (3/2)	**+**
	LNZR-1 (NZ_JGYJ00000000)	(3/2)	-
	QN1 (NZ_AJTH01000000)	(4/3); (3/2)	-
	SK14 (NZ_ACFR00000000)	(3/2); (3/2)	-
S. epidermidis	104_SEPI (NZ_JWFD0000000)	(3/2)	-
	1068_SEPI (NZ_JWEE00000000)	-	-
	114_SEPI (NZ_JWBR00000000)	-	-
	12142587 (NZ_AMSJ01000000)	-	-
	14.1.R1.SE (NZ_AGUC00000000)	-	-
	236_SEPI (NZ_JVOZ00000000)	(9/8)	-
	441_SEPI (NZ_JVGZ00000000)	-	-
	ATCC 12228 (NC_004461)	-	-
	BCM-HMP0060 (NZ_ACHE00000000)	-	-
	BVS058A4 (NZ_AGZV00000000)	(3/2)	-
	ENVP605 (NZ_LYVV00000000)	-	-
	FRI909 (NZ_AENR00000000)	(3/2)	-
	IS-250 (NZ_AJJR00000000)	-	-
	LRKNS062 (NZ_LWBW00000000)	-	-
	LRKNS075 (NZ_LWCV00000000)	-	-
	LRKNS080 (NZ_LXRP00000000)	-	-
	LRKNS083 (NZ_LXRU00000000)	-	-
	M23864:W2 (NZ_ADMU00000000)	-	-
	NIHLM003 (NZ_AKHB00000000)	-	-
	NIHLM015 (NZ_AKGZ00000000)	-	-
	NIHLM049 (NZ_AKGQ00000000)	-	-
	NIHLM087 (NZ_AKGK00000000)	-	-
	RP62A (NC_002976)	(4/3)	**+**
	Scl22 (NZ_ATDB00000000)	-	-
	SEI (NZ_CP009046)	-	-
	VCU037 (NZ_AFTY00000000)	(4/3); (5/4); (8/7)	**+**
	VCU041 (NZ_AHKX00000000)	-	-
	VCU071 (NZ_AGUB00000000)	-	-
	VCU105 (NZ_AFTZ00000000)	-	-
	VCU117 (NZ_AHLA00000000)	(10/9)	**+**
	VCU118 (NZ_AHLB00000000)	-	-
	VCU120 (NZ_AHLC00000000)	-	-
	VCU129 (NZ_AHLJ00000000)	-	-
	W23144 (NZ_ACJC0000000)	-	-
S. haemolyticus	109919 (NZ_CUFT01000001)	-	-
	112622 (NZ_CUGN01000001)	-	-
	115601 (NZ_CUHH01000000)	(3/2)	-
	124655 (NZ_CUGE01000001)	-	-
	1292_SHAE (NZ_JVVE01000069)	-	-
	140376 (NZ_CUHF01000001)	-	-
	164_SHAE (NZ_JVRQ01000059)	-	-
	1HT3 (NZ_LAKG01000022)	-	-
	51-03 (NZ_CUCR01000000)	-	-
	51-15 (NZ_CUFB01000000)	-	-
	51-17 (CUDD01000000)	-	-
	51-18 (CUDE01000000)	-	-
	51-37 (NZ_CUDV01000001)	-	-
	51-42 (NZ_CUDZ01000000)	-	-
	51-48 (NZ_CUEE01000000)	-	-
	51-50 (NZ_CUED01000000)	-	-
	51-51 (NZ_CUEI01000001)	-	-
	6_13 (NZ_CUEL01000000)	-	-
	6035 (NZ_CUFD01000000)	-	-
	643 (CUHP01000000)	(5/4); (4/3)	-
	92271 (NZ_CUFY01000001)	-	-
	96671 (NZ_CVRV01000001)	-	-
	C10A (NZ_JPRW01000000)	-	-
	CN1197 (NZ_CUEZ01000001)	-	-
	DNF00585 (NZ_JRNK01000002)	-	-
	F01 (NZ_CUWC01000001)	-	-
	JCSC1435 (AP006716)	-	-
	R1P1 (NZ_AJVA00000000)	-	-
	RIT283 (NZ_JFOJ01000002)	-	-
	Sh29/312/L2 (CP011116)	-	-
	W_75 (NZ_CUFQ01000000)	(23/22)	**+**
	W59 (NZ_CUFO01000001)	-	-

Table S1 (cont.). Coagulase-negative *Staphylococcus* genomes from strains of human origin analyzed in this work.

Species	Strains (access)	CRISPR-like arrangement (nº repeats/nº spacers)	*cas* genes
S. hominis	AS2 (NZ_LFKR00000000)	-	-
	C80 (NZ_ACRM00000000)	-	-
	RIT-PI-k (NZ_LHPB00000000)	-	-
	SK119 (NZ_ACLP00000000)	-	-
	VCU122 (NZ_AHLD00000000)	-	-
	ZBW5 (NZ_AKGC00000000)	(5/4)	-
S. lugdunensis	ACS-027-V-Sch2 (NZ_AGZW00000000)	(6/5); (5/4)	+
	FDAARGOS_141 (NZ_CP014022)	-	-
	FDAARGOS_143 (NZ_CP014023)	-	-
	HKU09-01 (NC_013893)	(7/6)	+
	M23590 (NZ_AEQA00000000)	(4/3); (3/2)	+
	N920143 (NC_017353)	(5/4)	+
	UCIM6116 (NZ_JBHB00000000)	-	-
	VCU139 (NZ_AHLK00000000)	-	-
	VCU148 (NZ_JIBR00000000)	-	-
S. massiliensis	CCUG 55927 (NZ_AKGE00000000)	(10/9); (3/2); (3/2); (3/2)	+
S. pettenkoferi	VCU012 (NZ_AGUA00000000)	-	-
S. saprophyticus	758_SSAP (NZ_JUUE00000000)	-	-
	ATCC 15305 (NC_007350)	-	-
	FDAARGOS_137 (NZ_CP014057)	-	-
	FDAARGOS_168 (NZ_CP014113)	-	-
	G764 (NZ_FKIN00000000)	-	-
	KACC 16562 (NZ_AHKB01000000)	-	-
	MF6029 (NZ_LSLC00000000)	-	-
	SU8 (NZ_JXBG00000000)	-	-
S. schleiferi	1360-13 (NZ_CP009470)	(37/36)	+
	2142-05 (NZ_CP009762)	(6/5)	+
	2317-03 (NZ_CP010309)	(19/18)	+
	5909-02 (NZ_CP009676)	(17/16)	+
	TSCC54 (NZ_AP014944)	(17/16); (16/15); (4/3)	+
S. sciuri	Z8 (NZ_JANE01000000)	(3/2)	-
S. simulans	ACS-120-V-Sch1 (NZ_AGZX00000000)	(3/2)	+
	CJ16 (NZ_LJSL00000000)	-	-
	FDAARGOS_124 (NZ_CP014016)	(11/10); (9/8)	+
S. succinus	CSM-77 (NZ_LUJH00000000)	-	-
S. warneri	1DB1 (NZ_LAKH00000000)	-	-
	691_SWAR (NZ_JUWX00000000)	(4/3)	+
	738_SWAR (NZ_JUVB00000000)	-	-
	A487 (NZ_CANQ00000000)	-	-
	FDAARGOS_151 (NZ_LORQ00000000)	-	-
	L37603 (NZ_ACPZ00000000)	-	-
	Lyso 1 2011 (NZ_JOPU00000000)	-	-
	Lyso 2 2011 (NZ_JOPV00000000)	-	-
	NGS-ED-1001 (NZ_JPOW00000000)	-	-
	VCU121 (NZ_AFEC00000000)	-	-
S. xylosus	C2A (NZ_LN554884)	-	-
	NJ (NZ_ANMR00000000)	-	-
	SMQ-121 (NZ_CP008724)	-	-
