# Supplementary material for: CRISPR-Cas Systems Features and the Gene-Reservoir Role of Coagulase-Negative Staphylococci
Source: Front Microbiol. 2017 Aug 15;8:1545. doi: 10.3389/fmicb.2017.01545 (PMC5559504; doi:10.3389/fmicb.2017.01545)
Supplement: Supplementary file 2 [file Table_2.PDF]

Table S2. Location of insertion sequences (IS) in the vicinity of the coagulase-negative *Staphylococcus* CRISPR loci described in this work

Strain	IS family	IS location (Genbank access, position)	IS distance from CRISPR (bp)
S. capitis CR01	IS6	NZ_HG737333, 169780-170508	11437
S. epidermidis RP64A	IS3	NC_002976, 2552263-2553523	34423
S. epidermidis VCU037	IS6	NZ_AFTY01000003, 56811-57592	35935
	IS3	NZ_AFTY01000003, 45879-46586	25003
S. lugdunensis ACS-027-V-Sch2	IS1182	NZ_KB373323, 282623-282859	11385
S. lugdunensis HKU09-01	IS1182	NC_013893, 9127-9379	20085
S. lugdunensis N920143	IS1182	NC_017353, 43100-43336	11367
S. massiliensis CCUG55927	IS6	NZ_JH815593, 1587760-1588233	46546
S. schleiferi 2317-03	IS30	NZ_CP010309, 1605301-1605635	11302
S. schleiferi TSCC54	IS6	NZ_AP014944, 49101-49890	16963
	IS6	NZ_AP014944, 54688-55418	11435
	IS1182	NZ_AP014944, 88052-88823	11043
	IS1183	NZ_AP014944, 88999-89738	11990
S. warneri 691_SWAR	IS3	NZ_JUWX01000029, 41451-41690	8196

*IS positions are given in accordance to their genome/scaffold’s sequences available from Genbank.
