# Supplementary material for: CRISPR-Cas Systems Features and the Gene-Reservoir Role of Coagulase-Negative Staphylococci
Source: Front Microbiol. 2017 Aug 15;8:1545. doi: 10.3389/fmicb.2017.01545 (PMC5559504; doi:10.3389/fmicb.2017.01545)
Supplement: Supplementary file 3 [file Table_3.PDF]

Table S3. Origins of spacer sequences from coagulase negative staphylococci analyzed in this work.

Strain	Spacer*	Spacer sequence	Spacer origin, Genbank ID
S. capitis CR01	scap01	CTAGATACCCAGAACAAAATAGGTCTAACGAAACAAT	unknown
	scap02	TCTATAAGTTCATTAATTCCGATACCTAGATTATCT	unknown
	scap03	AATTTTCTAATTCTATAAGTTCATTAATTCCGAT	unknown
	scap04	TAATAGTGTTGTTCTCTATTAAAAGATACAATCCTGT	unknown
	scap05	TAGAATGTTATTATCTAAGTGGTCGATGTATTCC	*Staphylococcus* phage phiIPLA-RODI, KP027446.1
	scap06	CTTAAAATCTAATTGCATTGTTATCAATTCCTTTA	unknown
	scap07	TCTGTAATGTATTCATTTAATGTAATCATAATTTTTTC	unknown
	scap08	TAGACCATTTACCTCATTATATTTATAGTCTTTATTA	unknown
	scap09	TTTTCTTTAACTGTTTTTACTGCCCATTTAATAGT	unknown
	scap10	ATAAACCCGTTCAATTCGTTATCTTTAAATTCTTG	unknown
	scap11	ACAACTTCGTCATCTTTCATCATTTCTCTTACATCA	unknown
	scap12	ATATTTCTTCCATGAATAACACCCTCCTTTTTTCTA	unknown
	scap13	AAGTTAACGGCATTACCTAATAAAAATATTTTAGG	unknown
	scap14	TCATCTTTCATGTCACTGATTAATTCATTTGTA	unknown
	scap15	GGTAATAGTTGCTCAATAGGTAATAAAACGTCGGT	unknown
S. capitis CR03	scap16	GCATAAAGTTTTGATATACACGATCGAATATGAGTT	unknown
	scap17	AGTGGTCCAGAATTAATACATGGTAAAGGAGTATATCAGTT	unknown
	scap18	TGAAGACCAATTAGAATCATATCGCGTCTTAGAAGAGTT	unknown
	scap19	CCTAAAATATTTTTATTAGGTAATGCCGTTAACT	unknown
	scap20	TGAAAAAAGGAGGGTGTTATTCATGGAAGAAATA	unknown
	scap21	TGATGTAAGAGAAATGATGAAAGATGACGAAGTTG	unknown
	scap22	CAAGAATTTAAAGATAACGAATTGAACGGGTTTA	unknown
	scap23	TAAGGAATTGATAACAATGCAATTAGATTTTAA	unknown
S. epidermidis RP62A	sepi01	GAGAATCAAGAAAAAATGTTCACGACCGTACTAGTTCTCGT	unknown
	sepi02	TCGATGTAACGTATGCAAATGACAATTATTACTAGTTCTCGT	*Staphylococcus* phage PH15, DQ834250.1
	sepi03	TTTGTACTGATGATTTATATACTTCGGCATACGTGTTCTCGT	*S lugdunensis* plasmid pT33G-1, KU882683.1 *S. aureus* plasmid pGO400, KT780705.1 *S. pseudintermedius* plasmid pKM01, KT373969.1
S. epidermidis VCU037	sepi04	CTTGTAAACTTACCAAACCACATGGCTGACGATGT	unknown
	sepi05	CAAGGTCCATTAGTAGGTCGTGAAAATGAAGTTAA	unknown
	sepi06	TAGTAAGTGATTTACATTATGACGGCATAGACGAACA	Staphylococcus phage 6ec, KJ804259.1
	sepi07	ATAAAAAAATTATTAAAAAGGATATGATTGAAATG	unknown
	sepi08	GCGAAAAGAATTTAAAATAATTGTTGGCTTTCAGAA	unknown
	sepi09	AGTCAATATAAAGACAATACTTTTTACGCTTATATT	unknown
	sepi10	GCATTATATGTTAGATATTGTCAACAGGTTTTTTT	unknown
	sepi11	TCTATCGTATTCCCTTTAGTTTCATCATTATTTGC	unknown
	sepi12	TGAATCATGTTCGCTATACGTTCCGGCTTTTCTT	unknown
	sepi13	TTTAATTTGTTTGCTCGATTGTGGGTTAAGCTAG	unknown
	sepi14	AATAAGGTCGTAAACTTGTTGGTAACTATCTTTTA	unknown
	sepi15	AGATTTCCTTACGACTAAGACCTAAAAATTCTTC	unknown
	sepi16	GCTCCTTTTATATATTTTATTTTGTCATTATTTAA	unknown
	sepi17	CAATAAATCCATTTTCTAATACATAATAATTTTTT	unknown

Table S3 (cont.). Origins of spacer sequences from coagulase negative staphylococci analyzed in this work.

**Strain**	**Spacer***	**Spacer sequence**	**Spacer origin, Genbank ID**
S. epidermidis VCU037	sepi18	TAGTAATAATTGTCATTTGCATACGTTACATCGAT	unknown
	sepi19	TAGTACGGTCGTGAACATTTTTTCTTGATTCTCT	unknown
S. haemolyticus W75	shae01	TATTCAAAATAAAAAGCCGACCTAAAAAAG	unknown
	shae02	CGATTATCCATGATTTTGGATATAAAATT	unknown
	shae03	CCCTTATTCCTTTACTATCTAATATGTCTTT	unknown
	shae04	TTCTAATTTCACTATGGACTAGCGAATTTA	unknown
	shae05	CACTTCGGATAAAATAAATACCCTACTCTA	unknown
	shae06	TAAGAGCATTATTAATTGTACCTAGCATT	unknown
	shae07	GGCAGGTTTACTTGGTTGTCCAGTGTCACT	unknown
	shae08	TGTACAACGAAGCAAAATTGCTAATACTAA	unknown
	shae09	AATCCGTCACCATACATATTTTGGTCTTT	unknown
	shae10	AAACATGTACAAAGACAAAATAAAAATTAT	*S. aureus* plasmid pUSA07-1-SUR24, CP014447.1
	shae11	CGACCGTGTATTTTTAGTTGATGAACGTAT	unknown
	shae12	ATGTTTGGTGATACACGACGCAAAGATGATA	unknown
	shae13	CATCTATATGGAATAGTTCATCACAACGTTA	unknown
	shae14	AAGAAGATATCATTGACTTGGGTAGTGATT	unknown
	shae15	GATGAACAAAAAGAATCTATTTACGGTAAG	unknown
	shae16	CCTCAGCACTTACAGTATCTTTATTTTCTT	*Staphylococcus* phage IME-SA4, KP735928.1
	shae17	TGCCACTAGGTTGTAGTTCTTCGCTAAAGTCA	unknown
	shae18	TATCGCTGCTAAACAATAAGGCGGTGTTGA	unknown
	shae19	AATCTTGTAAGTTCTCCTCTTTGAAATCAT	unknown
	shae20	TGAGCCTTTAACAAACACTTTCTGCTTGT	*S. aureus* pSAM12-0145, KU521355.1<br>*S. cohnii* plasmid pHK01, KC820816.1
	shae21	ATGATTTGGATTGGTTGTTAAGTTGGAATTA	*S. aureus* plasmid pUR4128, JQ861960.1
S. lugdunensis ACS-027-V-Sch2	slug01	TCTTGCCGTCCATAACATCTTTAGCTAATTGGAACA	unknown
	slug02	AGAAACAAACGGCGGATATGAGCATATAGTAAAATAT	unknown
	slug03	TAATGACACTTATGAATTGGTTAATAGAAATACAGAA	unknown
	slug04	TACAGTGATTTAATATATGGAAAAGTATACTGAGA	*S. aureus* plasmid pMI, AP017321.1<br>*S. epidermidis* plasmid SAP016A, GQ900381.1<br>*S. sciuri* plasmid pGFT2, AJ536194.1
	slug05	TAATAAAGGAGTTTTAACTATGTCAGTCTTAGATAAA	unknown
S. lugdunensis HLU09-01	slug06	TTCTGTATTTCTATTAACCAATTCATAAGTGTCATTA	unknown
	slug07	TTCATCATATCCTTTTATATAGTTTTTGTTTTGTT	unknown
	slug08	CCAGTTGCTATTTTATTTGTCAACCATTTTATTAA	unknown
	slug09	TAGAATGTTTAAACAAGGTGTTTCAAAACCTG	unknown
	slug10	AACATTTTATTTACGTCTGTTTTTGCCTCTACTAAGTAA	unknown
	slug11	TCGCTATTCAATAAGTTTTGTATTTGTTGTCTCATT	unknown
S. lugdunensis M23590	slug12	AAACCATGCCCAACAAAGCAATCCCTTATC	unknown
	slug13	ATAGAGATGAGAACGGTAATCTAATAGGTG	unknown
S. lugdunensis N920143	slug14	TAACACCTTTGATTTTATATCTTTTTCCATCAAAT	unknown
	slug15	CCAAATAATACTATTAATTTATCTGTTAATGCTGTGG	unknown
	slug16	TGGTAAATCTTCTACACTGTCGACTGGGTCTTCTA	unknown

Table S3 (cont.). Origins of spacer sequences from coagulase negative staphylococci analyzed in this work.

Strain	Spacer*	Spacer sequence	Spacer origin, Genbank ID
S. lugdunensis N920143	slug17	TGTTCCAATTAGCTAAAGATGTTATGGACGGCAAGA	unknown
S. massiliensis CCUG55927	smas01	GACGTGGTTAGTTACTTACGCAACATCAAT	unknown
	smas02	ATGCTCAAATAGAGTCGTGCAATCTTCCCA	unknown
	smas03	GCCATCTAAAAAATTAATAGTGTGTGTTT	unknown
	smas04	CAGAATAACCAGTTGTTTGACCAGTTACA	unknown
	smas05	TCTTGATACGCCTTTTTCATCTGTTTCATA	unknown
	smas06	TCTTGATACGCCTTTTTCATCTGTTTCATA	unknown
	smas07	TCTTGATACGCCTTTTTCATCTGTTTCATA	unknown
	smas08	GCATCTATTAAGGTTAAGAAACCGGATGCC	unknown
	smas09	TGTCGATAAGTTTGTTATTCGTTGTCATTT	unknown
S. schleiferi 1360-13	ssch01	CATAAATGTAATTTAATATCCTATCTTCTTG	unknown
	ssch02	CCCCATCAAACGAGCCATTTCATCTTGCGT	unknown
	ssch03	GCTCACACCTCCTAAAAACCAAATTCATCA	unknown
	ssch04	TTTTACATCAGGTTGAATTAAGTAGTTGCC	unknown
	ssch05	CGTAAATAACACAACCTTCGCGTCCACCATC	unknown
	ssch06	CAGTTTCTATATGTGATTTTTTCTCTATCC	*S. aureus* plasmid p140355, KY465818.1, *S. xylosus* plasmid pSX01, KP890694.1 *S. epidermidis* plasmid pSP01, KR230047.1 *S. schleiferi* 1360-13, CP009470.1
	ssch07	CCAAAGCTACCTTTAATTCCATGTTTCCTC	unknown
	ssch08	AAAAACGTAGGCAATGTAATGGTTGTTGAA	unknown
	ssch09	TAAATTTCAAACTTATATTTTGAGGTTCTGC	*S. epidermidis* plasmid p14-01514, KX520649.1 *S. aureus* plasmid pSAM12-0145, KU521355.1 *S. cohnii* plasmid pHK01, KC820816.1
	ssch10	GCTGTTGCTATCTATTCACTAACTCTTTTA	unknown
	ssch11	TTAAATGCATTTAATAAAAACCAATATTT	unknown
	ssch12	CAGCCACGTATGTTGTAACAGTGAATGTTT	unknown
	ssch13	AGGTGATATTGGAGTTGGTATTCCTAATA	unknown
	ssch14	CTGTATCGTATGGATAATAAATAACTTTCG	unknown
	ssch15	AAATGTTGCAAGGTGTTGAAGCAATGGCCG	unknown
	ssch16	TACAACAAGGTTCTAAAGAAGCTAGAGAAG	unknown
	ssch17	CAGCTTCAGGCGCTTCATGTTGTGCTTCTT	unknown
	ssch18	CGGCAAAAGCAATCATAGGGGGTGCAAAAT	unknown
	ssch19	TAAATCTTTTTCCATCAAATCATTAATAC	unknown
	ssch20	ATACCCTACTGAGGATGAATGGACAGAAGT	unknown
	ssch21	TGGCGCGAATTACGGCGGTGGATACGGTCA	unknown
	ssch22	CTTTCGTTTGCTTTGTAGCTTCAAACATTA	unknown
	ssch23	GTCATTGATGTCTAAGTAACTTTCTTCTGA	unknown
	ssch24	TGTTTAAAGTAAAGTTTGTCATTTTTGATT	unknown
	ssch25	GCAACGATTTGTCGGAAGAGAAGATAATCG	unknown
	ssch26	AAAGAGACGGAAATGGATATGTAAGGATTA	unknown
	ssch27	GAACGCAGAAAAGAACACGCCATAAGGTGC	unknown
	ssch28	CGTCGTGAATAATATAGTGCTTATTATTCG	unknown

Table S3 (cont.). Origins of spacer sequences from coagulase negative staphylococci analyzed in this work.

Strain	Spacer*	Spacer sequence	Spacer origin, Genbank ID
	ssch29	TGTTTCAATACCGAATTTATGAAGTGGTAT	unknown
	ssch30	GAGGTTGACCTGTATACTCTTCTATGTCGT	*Staphylococcus* phage SpT252, KX827370.1
	ssch31	ATAGTGGCATGATAGGTTCAAGTGTCCCTT	unknown
	ssch32	AGACTGGCGATGGTTCTGCGTTAAATACGT	unknown
	ssch33	CAGGTATAGCGCGTATAGATACCTTTTCTT	unknown
	ssch34	CGCCCCTCTAAAGATAATTCGATTCCCTCT	unknown
	ssch35	AAATAAAATAGCTTTAATTAATGGCTATTT	unknown
	ssch36	CATAAGCGACAGACTGTTGTTGATTTTCAT	unknown
	ssch37	TATCATAGAACAAGAGGATGACAGCTGGGT	unknown
S. schleiferi 2142-05	ssch38	GGGCTACTAATCTTTTGAACTCTGCATAGT	unknown
	ssch39	CTTCCTCTGTCGTTTCCTCCTTGTCACTTG	unknown
	ssch40	TTGTGATTCTTTATTTTGTCCGTATAATCT	unknown
	ssch41	AATGGCTTACATTAACAAATTCAACGAAAT	unknown
	ssch42	GTGTAACTCCTACTTGATTCGCAACACTCA	unknown
S. schleiferi 2317-03	ssch43	GGGCTACTAATCTTTTGAACTCTGCATAGT	unknown
	ssch44	TTGTGATTCTTTATTTTGTCCGTATAATCT	unknown
	ssch45	ACCAGCCACCAGGTTGGAACAGATACCCTA	unknown
	ssch46	GGATTTTCGCCCACAACTGACCTAGCCAGT	unknown
	ssch47	ACCAGCCACCAGGTTGGAACAGATACCCTA	unknown
	ssch48	GAATTCTTCTTCTGTAACAAATCCGCGCT	unknown
	ssch49	GTGTTACCTTCTCTTGTTCTAATAACTTAT	unknown
	ssch50	TTAGTTCAGGTCTAAGACGTAAATGCAGTA	unknown
	ssch51	AACGTATCCACCATAATTTATTTTTTACATT	unknown
	ssch52	CTACTTTACCGCAATCAGAAGTTAATATTG	unknown
	ssch53	CTAAACCTAAATAGTTTGTAGCAAGTTCAA	unknown
	ssch54	TGATCAGCTTCAAATTCAAGTTTTCCACCGT	unknown
	ssch55	TAGCTTTTCCACCTGTAAAATCTATTTTTG	unknown
	ssch56	TCTTTATCATGTTTAAAGTTGTAGAATACA	Phage 2638A, AY954954.1
	ssch57	CTAACAAATGTGAATATACGTCTAAAGTTG	unknown
	ssch58	TAGTTTTTTTATGGTCTGTAATCAACCTTA	unknown
	ssch59	GTGGAATTGTAGCAATCTTATTAGGTCTTG	unknown
	ssch60	AATGGCTTACATTAACAAATTCAACGAAAT	unknown
	ssch61	GTGGAACTCCTACTTGATTCGCAACACTCA	unknown
S. schleiferi 5909-02	ssch62	GGGCTACTAATCTTTTGAACTCTGCATAGT	unknown
	ssch63	ACCAGCCACCAGGTTGGAACAGATACCCTA	unknown
	ssch64	GAATTCTTCTTCTGTAACAAATCCGCGCT	unknown
	ssch65	TAGTGCCATTCATAATAGTTTCGAACCCA	unknown
	ssch66	TGATACGCATAATACAAATCCTTACCTAAAA	unknown
	ssch67	ATAGGCATACACCCGTAATCAAATTTGATA	unknown
	ssch68	GAAGAGATCTCAAGGTTTTTTAAAGATGAG	unknown
	ssch69	TAGGATAACCTTGTTTCAAAGAATTATGTTT	unknown
	ssch70	ATCAGTTGCCTCCTTTGTTATCGTAAAACA	unknown
	ssch71	TCTTTATCATGTTTAAAGTTGTAGAATACA	Phage 2638A, AY954954.1
	ssch72	CTAACAAATGTGAATATACGTCTAAAGTTG	unknown

Table S3 (cont.). Origins of spacer sequences from coagulase negative staphylococci analyzed in this work.

Strain	Spacer*	Spacer sequence	Spacer origin, Genbank ID
	ssch73	TAGTTTTTTTATGGTCTGTAATCAACCTTA	unknown
	ssch74	GTGGAATTGTAGCAATCTTATTAGGTCTTG	unknown
	ssch75	AATGGCTTACATTAACAAATTCAACGAAAT	unknown
	ssch76	CCAACAACTAAATAGTACCATAGGTGTTTC	unknown
	ssch77	GTGTAACTCCTACTTGATTCGCAACACTCA	unknown
S. schleiferi TSCC54	ssch78	ACTTCTCTCGCCATTTCTGCTAATTGTTCTACTTTG	unknown
	ssch79	ACTAGATACCCAGAACAAAATAGGTCTAACGAAA	unknown
	ssch80	TCTATAAGTTCATTAATTCCGATACCTAGATTATCT	unknown
	ssch81	TTTTTTCCACCCTTTCAGATCATCTATGATCTTG	unknown
	ssch82	AATTTTCTAATTCTATAAGTTCATTAATTCCGAT	unknown
	ssch83	TATACTATTTACATAATTTTTTATGTGTCTGTCTAC	unknown
	ssch84	TAATAGTGTTGTTCTCTATTAAAAGATACAATCCTGT	unknown
	ssch85	TCTGTAATGTATTCATTTAATGTAATCATAATTTTTTC	unknown
	ssch86	TAGACCATTTACCTCATTATATTTATAGTCTTTATTA	unknown
	ssch87	TTTTCTTTAACTGTTTTTACTGCCCATTTAATAGT	unknown
	ssch88	ATAAACCCGTTCAATTCGTTATCTTTAAATTCTTG	unknown
	ssch89	ACAACTTCGTCATCTTTCATCATTTCTCTTACATCA	unknown
	ssch90	ATATTTCTTCCATGAATAACACCCTCCTTTTTTCTA	unknown
	ssch91	AAGTTAACGGCATTACCTAATAAAAATATTTTAGG	unknown
	ssch92	TCATCTTTCATGTCACTGATTAATTCATTTGTA	unknown
	ssch93	GGTAATAGTTGCTCAATAGGTAATAAAACGTCGGT	unknown
	ssch94	CTTCTAAGACGCGATATGATTCTAATTGGTCTTCA	unknown
	ssch95	GATATACTCCTTTACCATGTATTAATTCTGGACCACT	unknown
	ssch96	CATATTCGATCGTGTATATCAAAACTTTATGC	unknown
	ssch97	CTTCCGAATCCATTTCAGCGCAATAAACA	unknown
	ssch98	GATTGATAGTTTGCGATTCTTGCAGTCATT	unknown
	ssch99	ACTCACTTGTAAATTCCTCCACTTGCTCTA	unknown
	ssch100	TCGTCATCAATCCATCGCCATATGCTTCTT	unknown
	ssch101	TGACAACCACGCTTTTAGCTTGCATATCAA	unknown
	ssch102	ATATTCTATTCAATTCGTTCTGCAACTTGT	unknown
	ssch103	TTACATAATCGTTTATGATTATTTCATGTT	unknown
	ssch104	CTGGAATAACCACAAAGCCAGAGTCAGTTT	unknown
	ssch105	CCATGATGTCTAAAAACACCGATTTACCAT	unknown
	ssch106	ACGTTAGATTTGCAGGTGTTAAGCACGGCT	unknown
	ssch107	TGTATGGGGTGCGGTCGACTCTACTATCA	unknown
	ssch108	GTAAATAATTCCGTTTGGACTCGTTACGTT	unknown
	ssch109	CCATCGCTTCTTTGCGTGAATTTGAAATAT	unknown
	ssch110	ATGTTTTTTTCATTAAAGCTACCAGTAATTC	unknown
	ssch111	AATTCGCCTTTATATTCAGGTTTCTTTTTT	unknown
S. simulans FDAARGOS_124	ssim01	CTTTCTAGTGCTAACTTGCGTGTATTATC	unknown
	ssim02	CAAAAGAACGTATTAATGGAAAACAATTA	unknown
	ssim03	TAGTGACAACAAAGCGAAAATCGAGATTA	unknown
	ssim04	AAGTGGGCGACAAGTATTTGAAAATTGCA	unknown
	ssim05	AAGTGGGCGACAAGTATTTAAAAATTGCA	unknown
	ssim06	AGCGGTAAACCCGTATTGATTAAAGCAAAG	unknown

Table S3 (cont.). Origins of spacer sequences from coagulase negative staphylococci analyzed in this work.

Strain	Spacer*	Spacer sequence	Spacer origin, Genbank ID
	ssim07	GATGCATCAAATGTTTTTACGCCCAATTCA	unknown
	ssim08	TGTGACTTACCTAAATCTAACATTTGATAT	unknown
	ssim09	ATTACGGAGGATGTCATCGGCTTTCTAACA	unknown
	ssim10	AAGTTTTGTGTGTTTCATGATACAGGAAA	unknown
	ssim11	TGTTTTAAATGCTTCTAAAGAAAGTGCTTT	unknown
	ssim12	ATGAACTCAATATCAGTGATAAGCGATTT	unknown
	ssim13	CAAGGTGAGCGTGTTTATCCGAACAAAAAG	unknown
	ssim14	TTGAACATATATGAAAGCTCACTGTAACCC	unknown
	ssim15	TTGTAGCATAACAACAAACTGTGCTCCTAA	unknown
	ssim16	AAAGCAGTATGAATGCAACAACAAAATCCT	unknown
	ssim17	AAAGTAATATCAATACAAATCGTATGGCAA	unknown
	ssim18	ATTAAGTTAGGCTTTGCAGATGAGAAATCA	unknown
S. warner 691_SWAR	swar01	GTGAAACATATCAAATGCTTAACTTAGGTAAATATC	unknown
	swar02	GTGAAACTCCTGCATACGAAAATACTTCAATCAGTGC	unknown
	swar03	GTGAAACGCTTTTTGAAAAATACAACTGAAGCTTTTA	unknown

*Some spacer sequences are identical: ssim04=ssim05, scap13=ssch91, scap03=ssch82, scap11=ssch89, scap45=scap47=ssch63, scap12=ssch90, scap15=ssch93, scap04=ssch84, scap08=ssch86, ssch58=ssch73, scap14=ssch92, scap02=ssch80, scap07=ssch85, scap03=ssch93, scap09=ssch87, ssch41=ssch60, ssch57=ssch72, ssch59=ssch74, ssch42=ssch77, ssch56=ssch71, ssch40=ssch44, ssch48=ssch64, ssch59=ssch74, ssch38=ssch43=ssch62, smas05=smas06=smas07. Total of unique sequences:194.
