# Supplementary material for: CRISPR-Cas Systems Features and the Gene-Reservoir Role of Coagulase-Negative Staphylococci
Source: Front Microbiol. 2017 Aug 15;8:1545. doi: 10.3389/fmicb.2017.01545 (PMC5559504; doi:10.3389/fmicb.2017.01545)
Supplement: Supplementary file 4 [file Image_1.PDF]

Strain: *Staphylococcus capitis* CR01
Genbank assess: NZ_CBUB000000000
CRISPR Location: scaffold NZ_HG737333.1, 181945-183054

181945	GATCGATAACTACCCCGAATAACAGGGGACGAGAAT	CTAGATACCCAGAACAAAATAGGTCTAACGAAACAAT	182017
182018	GATCGATAACTACCCCGAATAACAGGGGACGAGAAT	TCTATAAGTTCATTAATTCCGATACCTAGATTATCT	182089
182090	GATCGATAACTACCCCGAATAACAGGGGACGAGAAT	AATTTTCTAATTCTATAAGTTCATTAATTCCGAT	182159
182160	GATCGATAACTACCCCGAATAACAGGGGACGAGAAT	TAATAGTGTTGTTCTCTATTAAAAGATACAATCCTGT	182232
182233	GATCGATAACTACCCCGAATAACAGGGGACGAGAAT	TAGAATGTTATTATCTAAGTGGTCGATGTATTCC	182302
182303	GATCGATAACTACCCCGAATAACAGGGGACGAGAAT	CTTAAAATCTAATTGCATTGTTATCAATTCCTTTA	182373
182374	GATCGATAACTACCCCGAATAACAGGGGACGAGAAT	TCTGTAATGTATTCATTTAATGTAATCATAATTTTTTC	182447
182448	GATCGATAACTACCCCGAATAACAGGGGACGAGAAT	TAGACCATTTACCTCATTATATTTATAGTCTTTATTA	182520
182521	GATCGATAACTACCCCGAATAACAGGGGACGAGAAT	TTTTCTTTAACTGTTTTTACTGCCCATTTAATAGT	182591
182592	GATCGATAACTACCCCGAATAACAGGGGACGAGAAT	ATAAACCCGTTCAATTCGTTATCTTTAAATTCTTG	182662
182663	GATCGATAACTACCCCGAATAACAGGGGACGAGAAT	ACAACTTCGTCATCTTTCATCATTTCTCTTACATCA	182734
182735	GATCGATAACTACCCCGAATAACAGGGGACGAGAAT	ATATTTCTTCCATGAATAACACCCTCCTTTTTTCTA	182806
182807	GATCGATAACTACCCCGAATAACAGGGGACGAGAAT	AAGTTAACGGCATTACCTAATAAAAATATTTTAGG	182877
182878	GATCGATAACTACCCCGAATAACAGGGGACGAGAAC	TCATCTTTCATGTCACTGATTAATTCATTTGTA	182946
182947	GATCGATAACTACCCCGAATAACAGGGGACGAGAAC	GGTAATAGTTGCTCAATAGGTAATAAAACGTCGGT	183017
183018	GATCGATAACTATCCCGAATAACAGGGGACAGAGTG		183053

Strain: *Staphylococcus capitis* CR03
Genbank assess: NZ_CVUF00000000
CRISPR Location: scaffold NZ_CVUF01000007.1, 50247-50494 and 59216-59608

50247	CTCGTCCCCTCTTCTACGGGGTAGTTATCGAAT	GCATAAAGTTTTGATATACACGATCGAATATGAGTT	50315
50316	CTCGTCCCCTCTTCTACGGGGTAGTTATCGAAT	AGTGGTCCAGAATTAATACATGGTAAAGGAGTATATCAGTT	50389
50390	CTCGTCCCCTCTTCTACGGGGGTAGTTATCGAA	TGAAGACCAATTAGAATCATATCGCGTCTTAGAAGAGTT	50461
50462	CTCGTCCCCTCTTCTACGGGGTAGTTATCGAAT		50494

59216	ACACTCTGTCCCCTGTTATTCGGGATAGTTATCGATC	CCTAAAATATTTTTATTAGGTAATGCCGTTAACT	59286
59287	TATTCTCGTCCCCTGTTATTCGGGGTAGTTATCGATC	TAGAAAAAAGGAGGGTGTTATTCATGGAAGAAATA	59358
59359	TATTCTCGTCCCCTGTTATTCGGGGTAGTTATCGATC	TGATGTAAGAGAAATGATGAAAGATGACGAAGTTG	59430
59431	TATTCTCGTCCCCTGTTATTCGGGGTAGTTATCGATC	CAAGAATTTAAAGATAACGAATTGAACGGGTTTA	59501
59502	TATTCTCGTCCCCTGTTATTCGGGGTAGTTATCGATC	TAAGGAATTGATAACAATGCAATTAGATTTTAA	59571
59572	GATTCTCGTCCCCTGTTATTCGGGGGTAGTTATCGAT		59608

Strain: *Staphylococcus epidermidis* RP62A
Genbank assess: NC_002976
CRISPR Location: 2517628-2517868

2517628	CCCCTTTTCTTCGGGGTGGCTATCGATCA	GAGAATCAAGAAAAAATGTTCACGACCGTACTAGTTCTCGT	2517697
2517698	CCCCTTTTCTTCGGGGTGGGTATCGATCA	TCGATGTAACGTATGCAAATGACAATTATTACTAGTTCTCGT	2517768
2517769	CCCCTTTTCTTCGGGGTGGGTATCGATCC	TTTGTACTGATGATTTATATACTTCGGCATACGTGTTCTCGT	2517839
2517840	CCCCTTTTCTTCGGGGTGGGTATCGATCC		2517868

Strain: *Staphylococcus epidermidis* VCU037
Genbank assess: NZ_AFTY00000000
CRISPR Location: scaffold NZ_AFTY01000003.1, 20340-20876

20340	CACTCTGCCCCCTTTTCTTCGGGGTAGTTATCGATC	CTTGTAAACTTACCAAACCACATGGCTGACGATGT	20410
20411	ATTCTCGTCCCCTTTTCTTCGGGGTGGTTATCGATC	CAAGGTCCATTAGTAGGTCGTGAAAATGAAGTTAA	20481
20482	GTTCTCGTCCCCTTTTCTTCGGGGTGGTTATCGATC	TAGTAAGTGATTTACATTATGACGGCATAGACGAACA	20554
20555	GTTCTCGTCCCCTTTTCTTCGGGGTGGTTATCGATC	ATAAAAAAATTATTAAAAAGGATATGATTGAAATG	20625
20626	GTTCTCGTCCCCTTTTCTTCGGGGTGGTTATCGATC	GCGAAAAGAATTTAAAATAATTGTTGGCTTTCAGAA	20697
20698	GTTCTCGTCCCCTTTTCTTCGGGGTGGTTATCGATC	AGTCAATATAAAGACAATACTTTTTACGCTTATATT	20769
20770	GTTCTCGTCCCCTTTTCTTCGGGGTGGTTATCGATC	GCATTATATGTTAGATATTGTCAACAGGTTTTTTT	20840
20841	GTTCTCGTCCCCTTTTCTTCGGGGTGGTTATCGATC		20876

Figure S1. Sequence structure of CRISPR loci discovered in coagulase-negative *Staphylococcus* strains from human origin described in this work.

Strain: *Staphylococcus epidermidis* VCU117
Genbank assess: NZ_AHLA00000000
CRISPR Location: scaffold NZ_AHLA01000017.1, 12780-13450

12780	GATCGATACCCACCCCGAAGAAAAGGGGACGAGAAC	TCTATCGTATTCCCTTTAGTTTCATCATTATTTGC	12850
12851	GATCGATACCCACCCCGAAGAAAAGGGGACGAGAAC	TGAATCATGTTCGCTATACGTTCCGGCTTTTCTT	12920
12921	GATCGATACCCACCCCGAAGAAAAGGGGACGAGAAC	TTTAATTTGTTTGCTCGATTGTGGGTTAAGCTAG	12990
12991	GATCGATACCCACCCCGAAGAAAAGGGGACGAGAAC	AATAAGGTCGTAAACTTGTTGGTAACTATCTTTTA	13061
13062	GATCGATACCCACCCCGAAGAAAAGGGGACGAGAAC	AGATTTCCTTACGACTAAGACCTAAAAATTCTTC	13131
13132	GATCGATACCCACCCCGAAGAAAAGGGGACGAGAAC	GCTCCTTTTATATATTTTATTTTGTCATTATTTAA	13202
13203	GATCGATACCCACCCCGAAGAAAAGGGGACGAGAAC	CAATAAATCCATTTTCTAATACATAATAATTTTTT	13273
13274	GATCGATACCCACCCCGAAGAAAAGGGGACGAGAAC	TAGTAATAATTGTCATTTGCATACGTTACATCGAT	13344
13345	GATCGATACCCACCCCGAAGAAAAGGGGACGAGAAC	TAGTACGGTCGTGAACATTTTTTCTTGATTCTCT	13414
13415	GATCGATAGCCACCCCGAAGAAAAGGGGGCAGAGTG		13450

Strain: *Staphylococcus haemolyticus* W75
Genbank assess: NZ_CUFQ01000000
CRISPR Location: scaffold CUFQ01000030.1, 17332-18719

17332	ATATACCTCATATCTAAAATTACAGAGTACTAAAAC	TATTCAAAATAAAAAGCCGACCTAAAAAAG	17397
17398	ATATACCTCATACCTAAAATTACAGAGTACTAAAAC	CGATTATCCATGATTTTGGATATAAAATT	17462
17463	GTCTACCTCATACCTAAAATTACAGAGTACTAAAAC	CCCTTATTCCTTTACTATCTAATATGTCTTT	17529
17530	GTCTACCTCATACCTAAAATTACAGAGTACTAAAAC	TTCTAATTTCACTATGGACTAGCGAATTTA	17595
17596	GTCTACCTCATACCTAAAATTACAGAGTACTAAAAC	CACTTCGGATAAAATAAATACCCTACTCTA	17661
17662	GTCTACCTCATACCTAAAATTACAGAGTACTAAAAC	TAAGAGCATTATTAATTGTACCTAGCATT	17726
17727	GTCTACCTCATACCTAAAATTACAGAGTACTAAAAC	GGCAGGTTTACTTGGTTGTCCAGTGTCACT	17792
17793	GTCTACCTCATACCTAAAATTACAGAGTACTAAAAC	TGTACAACGAAGCAAAATTGCTAATACTAA	17858
17859	GTCTACCTCATACCTAAAATTACAGAGTACTAAAAC	AATCCGTCACCATACATATTTTGGTCTTT	17923
17924	GTCTACCTCATACCTAAAATTACAGAGTACTAAAAC	AAACATGTACAAAGACAAAATAAAAATTAT	17989
17990	GTCTACCTCATACCTAAAATTACAGAGTACTAAAAC	CGACCGTGTATTTTTAGTTGATGAACGTAT	18055
18056	GTCTACCTCATACCTAAAATTACAGAGTACTAAAAC	ATGTTTGGTGATACACGACGCAAAGATGATA	18122
18123	GTCTACCTCATACCTAAAATTACAGAGTACTAAAAC	CATCTATATGGAATAGTTCATCACAACGTTA	18189
18190	GTCTACCTCATATCTAAAATTACAGAGTACTAAAAC	AAGAAGATATCATTGACTTGGGTAGTGATT	18255
18256	GTCTACCTCATACCTAAAATTACAGAGTACTAAAAC	GATGAACAAAAAGAATCTATTTACGGTAAG	18321
18322	GTCTACCTCATACCTAAAATTACAGAGTACTAAAAC	CCTCAGCACTTACAGTATCTTTATTTTCTT	18387
18388	GTCTACCTCATACCTAAAATTACAGAGTACTAAAAC	TGCCACTAGGTTGTAGTTCTTCGCTAAAGTCA	18455
18456	GTCTACCTCATACCTAAAATTACAGAGTACTAAAAC	TATCGCTGCTAAACAATAAGGCGGTGTTGA	18521
18522	GTCTACCTCATACCTAAAATTACAGAGTACTAAAAC	AATCTTGTAAGTTCTCCTCTTTGAAATCAT	18587
18588	GTCTACCTCATACCTAAAATTACAGAGTACTAAAAC	TGAGCCTTTAACAAACACTTTCTGCTTGT	18652
18653	GTCTACCTCATACCTAAAATTACAGAGTACTAAAAC	ATGATTTGGATTGGTTGTTAAGTTGGAATTA	18719

Strain: *Staphylococcus lugdunensis* ACS-027-V-Sch2
Genbank assess: NZ_AGZW00000000
CRISPR Location: scaffold NZ_KB373323.1, 262089-262486

262089	CTTTTTGTCCCCTTTTATACGGGGTATTCTCTGATT	TCTTGCCGTCCATAACATCTTTAGCTAATTGGAACA	262160
262161	GTTCTCGTCCCCTTTTATACGGGGTATTCTCTGATT	AGAAACAAACGGCGGATATGAGCATATAGTAAAATAT	262233
262234	GTTCTCGTCCCCTTTTATACGGGGTATTCTCTGATT	TAATGACACTTATGAATTGGTTAATAGAAATACAGAA	262306
262307	GTTCTCGTCCCCTTTTATACGGGGTATTCTCTGATT	TACAGTGATTTAATATATGGAAAAGTATACTGAGA	262377
262378	GTTCTCGTCCCCTTTTATACGGGGTATTCTCTGATT	TAATAAAGGAGTTTTAACTATGTCAGTCTTAGATAAA	262450
262451	GTTCTCGTCCCCTTTTATACGGGGTATTCTCTGATT		262486

Strain: *Staphylococcus lugdunensis* HKU09-01
Genbank assess: NC_013893
CRISPR Location: 29464-29929

29464	AATCAGAGAATACCCCGTATAAAAGGGGACGAGAAC	TTCTGTATTTCTATTAACCAATTCATAAGTGTCATTA	29536
29537	AATCAGAGAATACCCCGTATAAAAGGGGACGAGAAC	TTCATCATATCCTTTTATATAGTTTTTGTTTTGTT	29607
29608	AATCAGAGAATACCCCGTATAAAAGGGGACGAGAAC	CCAGTTGCTATTTTATTTGTCAACCATTTTATTAA	29678
29679	AATCAGAGAATACCCCGTATAAAAGGGGACGAGAAC	TAGAATGTTTAAACAAGGTGTTTCAAAACCTG	29746
29747	AATCAGAGAATACCCCGTATAAAAGGGGACGAGAAC	AACATTTTATTTACGTCTGTTTTTGCCTCTACTAAGTAA	29821
29822	AATCAGAGAATACCCCGTATAAAAGGGGACGAGAAC	TCGCTATTCAATAAGTTTTGTATTTGTTGTCTCATT	29893
29894	AATCAGAGAATACCCCGTATAAAAGGGGACAAAAAG		29929

Figure S1. Sequence structure of CRISPR loci discovered in coagulase-negative *Staphylococcus* strains from human origin described in this work.

Strain: *Staphylococcus lugdunensis* M23590
Genbank assess: NZ_AEQA00000000
CRISPR Location: scaffold NZ_GL622352.1, 733446-733613

733446	GTATCATTTATACCTAAAATTACAGAGTACTAAAAC	AAACCATGCCCAACAAAGCAATCCCTTATC	733511
733512	GTATCACTTATACCTAAAATTACAGAGTACTAAAAC	ATAGAGATGAGAACGGTAATCTAATAGGTG	733577
733578	GTATCACTTATACCTAAAATTACAGAGTACTAAAAC		733613

Strain: *Staphylococcus lugdunensis* N920143
Genbank assess: NC_017353
CRISPR Location: 63455-63777

63455	AATCAGAGAATACCCCGTATAAAAGGGGACGAGAAC	TAACACCTTTGATTTTATATCTTTTTCCATCAAAT	63525
63526	AATCAGAGAATACCCCGTATAAAAGGGGACGAGAAC	CCAAATAATACTATTAATTTATCTGTTAATGCTGTGG	63598
63599	AATCAGAGAATACCCCGTATAAAAGGGGACGAGAAC	TGGTAAATCTTCTACACTGTCGACTGGGTCTTCTA	63669
63670	AATCAGAGAATACCCCGTATAAAAGGGGACGAGAAC	TGTTCCAATTAGCTAAAGATGTTATGGACGGCAAGA	63741
63742	AATCAGAGAATACCCCGTATAAAAGGGGACAAAAAG		63777

Strain: *Staphylococcus massiliensis* CCUG 55927
Genbank assess: NZ_AKGE00000000
CRISPR Location: scaffold NZ_JH815593.1, 1534549-1535176

1534549	CTTGAACTTATACCTAAAATTACAGAGTACTAAAAC	GACGTGGTTAGTTACTTACGCAACATCAAT	1534614
1534615	GAATCACTTATACCTAAAATTATAGAGTACTAAAAC	ATGCTCAAATAGAGTCGTGCAATCTTCCCA	1534680
1534681	GAATCACTTATACCTAAAAATACAGAGTACTAAAAC	GCCATCTAAAAAATTAATAGTGTGTGTTT	1534745
1534746	GAATCACTTATACCTAAAATTACAGAGTACTAAAAC	CAGAATAACCAGTTGTTTGACCAGTTACA	1534810
1534811	GAATCACTTATACCTAAAATTACAGAGTACTAAAAC	TCTTGATACGCCTTTTTCATCTGTTTCATA	1534876
1534877	GAATCACTTATACCTAAAATTACAGAGTACTAAAAC	TCTTGATACGCCTTTTTCATCTGTTTCATA	1534942
1534943	GAATCACTTATACCTAAAATTACAGAGTACTAAAAC	TCTTGATACGCCTTTTTCATCTGTTTCATA	1535008
1535009	GAATCACTTATACCTAAAATTACAGAGTACTAAAAC	GCATCTATTAAGGTTAAGAAACCGGATGCC	1535074
1535075	GAATCACTTATACCTAAAATTACAGAGTACTAAAAC	TGTCGATAAGTTTGTTATTCGTTGTCATTT	1535140
1535141	GAATCACTTATACCTAAAATTACAGAGTACTAAAAC		1535176

Strain: *Staphylococcus schleiferi* 1360-13
Genbank assess: NZ_CP009470
CRISPR Location: 1478814-1481291

1478814	GACTAGTATTCATTTAAAATTGTAGAGTGCTAAAAC	CATAAATGTAATTTAATATCCTATCTTCTTG	1478880
1478881	GTTTCATTTATACCTAAAATTACAGAGTACTAAAAC	CCCCATCAAACGAGCCATTTCATCTTGCGT	1478946
1478947	GTTTCATTTATACCTAAAATTACAGAGTACTAAAAC	GCTCACACCTCCTAAAAACCAAATTCATCA	1479012
1479013	GTTTCATTTATACCTAAAATTACAGAGTACTAAAAC	TTTTACATCAGGTTGAATTAAGTAGTTGCC	1479078
1479079	GTTTCATTTATACCTAAAATTACAGAGTACTAAAAC	CGTAAATAACACAACCTTCGCGTCCACCATC	1479145
1479146	GTTTCATTTATACCTAAAATTACAGAGTACTAAAAC	CAGTTTCTATATGTGATTTTTTCTCTATCC	1479211
1479212	GTTTCATTTATACCTAAAATTACAGAGTACTAAAAC	CCAAAGCTACCTTTAATTCCATGTTTCCTC	1479277
1479278	GTTTCATTTATACCTAAAATTACAGAGTACTAAAAC	AAAAACGTAGGCAATGTAATGGTTGTTGAA	1479343
1479344	GTTTCATTTATACCTAAAATTACAGAGTACTAAAAC	TAAATTTCAAACTTATATTTTGAGGTTCTGC	1479410
1479411	GTTTCATTTATACCTAAAATTACAGAGTACTAAAAC	GCTGTTGCTATCTATTCACTAACTCTTTTA	1479476
1479477	GTTTCATTTATACCTAAAATTACAGAGTACTAAAAC	TTAAATGCATTTAATAAAAACCAATATTT	1479541
1479542	GTTTCATTTATACCTAAAATTACAGAGTACTAAAAC	CAGCCACGTATGTTGTAACAGTGAATGTTT	1479607
1479608	GTTTCATTTATACCTAAAATTACAGAGTACTAAAAC	AGGTGATATTGGAGTTGGTATTCCTAATA	1479672
1479673	GTTTCATTTATACCTAAAATTACAGAGTACTAAAAC	CTGTATCGTATGGATAATAAATAACTTTCG	1479738
1479739	GTTTCATTTATACCTAAAATTACAGAGTACTAAAAC	AAATGTTGCAAGGTGTTGAAGCAATGGCCG	1479804
1479805	GTTTCATTTATACCTAAAATTACAGAGTACTAAAAC	TACAACAAGGTTCTAAAGAAGCTAGAGAAG	1479870
1479871	GTTTCATTTATACCTAAAATTACAGAGTACTAAAAC	CAGCTTCAGGCGCTTCATGTTGTGCTTCTT	1479936
1479937	GTTTCATTTATACCTAAAATTACAGAGTACTAAAAC	CGGCAAAAGCAATCATAGGGGGTGCAAAAT	1480002
1480003	GTTTCATTTATACCTAAAATTACAGAGTACTAAAAC	TAAATCTTTTTCCATCAAATCATTAATAC	1480067
1480068	GTTTCATTTATACCTAAAATTACAGAGTACTAAAAC	ATACCCTACTGAGGATGAATGGACAGAAGT	1480133
1480134	GTTTCATTTATACCTAAAATTACAGAGTACTAAAAC	TGGCGCGAATTACGGCGGTGGATACGGTCA	1480199
1480200	GTTTCATTTATACCTAAAATTACAGAGTACTAAAAC	CTTTCGTTTGCTTTGTAGCTTCAAACATTA	1480265
1480266	GTTTCATTTATACCTAAAATTACAGAGTACTAAAAC	GTCATTGATGTCTAAGTAACTTTCTTCTGA	1480331
1480332	GTTTCATTTATACCTAAAATTACAGAGTACTAAAAC	TGTTTAAAGTAAAGTTTGTCATTTTTGATT	1480397
1480398	GTTTCATTTATACCTAAAATTACAGAGTACTAAAAC	GCAACGATTTGTCGGAAGAGAAGATAATCG	1480463
1480464	GTTTCATTTATACCTAAAATTACAGAGTACTAAAAC	AAAGAGACGGAAATGGATATGTAAGGATTA	1480529
1480530	GTTTCATTTATACCTAAAATTACAGAGTACTAAAAC	GAACGCAGAAAAGAACACGCCATAAGGTGC	1480595
1480596	GTTTCATTTATACCTAAAATTACAGAGTACTAAAAC	CGTCGTGAATAATATAGTGCTTATTATTCG	1480661
1480662	GTTTCATTTATACCTAAAATTACAGAGTACTAAAAC	TGTTTCAATACCGAATTTATGAAGTGGTAT	1480727

Figure S1. Sequence structure of CRISPR loci discovered in coagulase-negative *Staphylococcus* strains from human origin described in this work.

1480728	GTTTCATTTATACCTAAAATTACAGAGTACTAAAAC	GAGGTTGACCTGTATACTCTTCTATGTCGT	1480793
1480794	GTTTCATTTATACCTAAAATTACAGAGTACTAAAAC	ATAGTGGCATGATAGGTTCAAGTGTCCCTT	1480859
1480860	GTTTCATTTATACCTAAAATTACAGAGTACTAAAAC	AGACTGGCGATGGTTCTGCGTTAAATACGT	1480925
1480926	GTTTCATTTATACCTAAAATTACAGAGTACTAAAAC	CAGGTATAGCGCGTATAGATACCTTTTCTT	1480991
1480992	GTTTCATTTATACCTAAAATTACAGAGTACTAAAAC	CGCCCCTCTAAAGATAATTCGATTCCCTCT	1481057
1481058	GTTTCATTTATACCTAAAATTACAGAGTACTAAAAC	AAATAAAATAGCTTTAATTAATGGCTATTT	1481123
1481124	GTTTCATTTATACCTAAAATTACAGAGTACTAAAAC	CATAAGCGACAGACTGTTGTTGATTTTCAT	1481189
1481190	GTTTCATTTATACCTAAAATTACAGAGTACTAAAAC	TATCATAGAACAAGAGGATGACAGCTGGGT	1481255
1481256	GTTTCATTTATACCTAAAATTACAGAGTACTAAAAC		1481291

Strain: *Staphylococcus schleiferi* 2142-05
Genbank assess: NZ_CP009762
CRISPR Location: 1478868-1479233

1478868	AATAAGTATTCATTTAAAATTACAAAGTACTAAAAC	GGGCTACTAATCTTTTGAACTCTGCATAGT	1478933
1478934	GTTTCATTTATACCTAAAATTACAGAGTACTAAAAC	CTTCCTCTGTCGTTTCCTCCTTGTCACTTG	1478999
1479000	GTTTCATTTATACCTAAAATTACAGAGTACTAAAAC	TTGTGATTCTTTATTTTGTCCGTATAATCT	1479065
1479066	GTTTCATTTATACCTAAAATTACAGAGTACTAAAAC	AATGGCTTACATTAACAAATTCAACGAAAT	1479131
1479132	GTTTCATTTATACCTAAAATTACAGAGTACTAAAAC	GTGTAACTCCTACTTGATTCGCAACACTCA	1479197
1479198	GTTTCATTTATACCTAAAATTACAGAGTACTAAAAC		1479233

Strain: *Staphylococcus schleiferi* 2317-03
Genbank assess: NZ_CP010309
CRISPR Location: 1585815-1587105

1585815	AATAAGTATTCATTTAAAATTACAAAGTACTAAAAC	GGGCTACTAATCTTTTGAACTCTGCATAGT	1585880
1585881	GTTTCATTTATACCTAAAATTACAGAGTACTAAAAC	TTGTGATTCTTTATTTTGTCCGTATAATCT	1585946
1585947	GTTTCATTTATACCTAAAATTACAGAGTACTAAAAC	ACCAGCCACCAGGTTGGAACAGATACCCTA	1586012
1586013	GTTTCATTTATACCTAAAATTACAGAGTACTAAAAC	GGATTTTCGCCCACAACTGACCTAGCCAGT	1586078
1586079	GTTTCATTTATACCTAAAATTACAGAGTACTAAAAC	ACCAGCCACCAGGTTGGAACAGATACCCTA	1586144
1586145	GTTTCATTTATACCTAAAATTACAGAGTACTAAAAC	GAATTCTTCTTCTGTAACAAATCCGCGCT	1586209
1586210	GTTTCATTTATACCTAAAATTACAGAGTACTAAAAC	GTGTTACCTTCTCTTGTTCTAATAACTTAT	1586275
1586276	GTTTCATTTATACCTAAAATTACAGAGTACTAAAAC	TTAGTTCAGGTCTAAGACGTAAATGCAGTA	1586341
1586342	GTTTCATTTATACCTAAAATTACAGAGTACTAAAAC	AACGTATCCACCATAATTTATTTTTTACATT	1586408
1586409	GTTTCATTTATACCTAAAATTACAGAGTACTAAAAC	CTACTTTACCGCAATCAGAAGTTAATATTG	1586474
1586475	GTTTCATTTATACCTAAAATTACAGAGTACTAAAAC	CTAAACCTAAATAGTTTGTAGCAAGTTCAA	1586540
1586541	GTTTCATTTATACCTAAAATTACAGAGTACTAAAAC	TGATCAGCTTCAAATTCAAGTTTTCCACCGT	1586607
1586608	GTTTCATTTATACCTAAAATTACAGAGTACTAAAAC	TAGCTTTTCCACCTGTAAAATCTATTTTTG	1586673
1586674	GTTTCATTTATACCTAAAATTACAGAGTACTAAAAC	TCTTTATCATGTTTAAAGTTGTAGAATACA	1586739
1586740	GTTTCATTTATACCTAAAATTACAGAGTACTAAAAC	CTAACAAATGTGAATATACGTCTAAAGTTG	1586805
1586806	GTTTCATTTATACCTAAAATTACAGAGTACTAAAAC	TAGTTTTTTTATGGTCTGTAATCAACCTTA	1586871
1586872	GTTTCATTTATACCTAAAATTACAGAGTACTAAAAC	GTGGAATTGTAGCAATCTTATTAGGTCTTG	1586937
1586938	GTTTCATTTATACCTAAAATTACAGAGTACTAAAAC	AATGGCTTACATTAACAAATTCAACGAAAT	1587003
1587004	GTTTCATTTATACCTAAAATTACAGAGTACTAAAAC	GTGGAACTCCTACTTGATTCGCAACACTCA	1587069
1587070	GTTTCATTTATACCTAAAATTACAGAGTACTAAAAC		1587105

Strain: *Staphylococcus schleiferi* 5909-02
Genbank assess: NZ_CP009676
CRISPR Location: 1544989-1546080

1544989	AATAAGTATTCATTTAAAATTACAAAGTACTAAAAC	GGGCTACTAATCTTTTGAACTCTGCATAGT	1545054
1545055	GTTTCATTTATACCTAAAATTACAGAGTACTAAAAC	ACCAGCCACCAGGTTGGAACAGATACCCTA	1545120
1545121	GTTTCATTTATACCTAAAATTACAGAGTACTAAAAC	GAATTCTTCTTCTGTAACAAATCCGCGCT	1545185
1545186	GTTTCATTTATACCTAAAATTACAGAGTACTAAAAC	TAGTGCCATTCATAATAGTTTCGAACCCA	1545250
1545251	GTTTCATTTATACCTAAAATTACAGAGTACTAAAAC	TGATACGCATAATACAAATCCTTACCTAAAA	1545317
1545318	GTTTCATTTATACCTAAAATTACAGAGTACTAAAAC	ATAGGCATACACCCGTAATCAAATTTGATA	1545383
1545384	GTTTCATTTATACCTAAAATTACAGAGTACTAAAAC	GAAGAGATCTCAAGGTTTTTTAAAGATGAG	1545449
1545450	GTTTCATTTATACCTAAAATTACAGAGTACTAAAAC	TAGGATAACCTTGTTTCAAAGAATTATGTTT	1545516
1545517	GTTTCATTTATACCTAAAATTACAGAGTACTAAAAC	ATCAGTTGCCTCCTTTGTTATCGTAAAACA	1545582
1545583	GTTTCATTTATACCTAAAATTACAGAGTACTAAAAC	TCTTTATCATGTTTAAAGTTGTAGAATACA	1545648
1545649	GTTTCATTTATACCTAAAATTACAGAGTACTAAAAC	CTAACAAATGTGAATATACGTCTAAAGTTG	1545714
1545715	GTTTCATTTATACCTAAAATTACAGAGTACTAAAAC	TAGTTTTTTTATGGTCTGTAATCAACCTTA	1545780
1545781	GTTTCATTTATACCTAAAATTACAGAGTACTAAAAC	GTGGAATTGTAGCAATCTTATTAGGTCTTG	1545846
1545847	GTTTCATTTATACCTAAAATTACAGAGTACTAAAAC	AATGGCTTACATTAACAAATTCAACGAAAT	1545912
1545913	GTTTCATTTATACCTAAAATTACAGAGTACTAAAAC	CCAACAACTAAATAGTACCATAGGTGTTTC	1545978
1545979	GTTTCATTTATACCTAAAATTACAGAGTACTAAAAC	GTGTAACTCCTACTTGATTCGCAACACTCA	1546044
1546045	GTTTCATTTATACCTAAAATTACAGAGTACTAAAAC		1546080

Figure S1. Sequence structure of CRISPR loci discovered in coagulase-negative *Staphylococcus* strains from human origin described in this work.

Strain: *Staphylococcus schleiferi* TSCC54
Genbank assess: NZ_AP014944
CRISPR Location: 66853-68031, 76758-77009 and 1549038-1550061

66853	GATCGATAACTACCCCGAATAACAGGGGACGAGAAT	ACTTCTCTCGCCATTTCTGCTAATTGTTCTACTTTG	66924
66925	GATCGATAACTACCCCGAATAACAGGGGACGAGAAT	ACTAGATACCCAGAACAAAATAGGTCTAACGAAA	66994
66995	GATCGATAACTACCCCGAATAACAGGGGACGAGAAT	TCTATAAGTTCATTAATTCCGATACCTAGATTATCT	67066
67067	GATCGATAACTACCCCGAATAACAGGGGACGAGAAT	TTTTTTCCACCCTTTCAGATCATCTATGATCTTG	67136
67137	GATCGATAACTACCCCGAATAACAGGGGACGAGAAT	AATTTTCTAATTCTATAAGTTCATTAATTCCGAT	67206
67207	GATCGATAACTACCCCGAATAACAGGGGACGAGAAT	TATACTATTTACATAATTTTTTATGTGTCTGTCTAC	67278
67279	GATCGATAACTACCCCGAATAACAGGGGACGAGAAT	TAATAGTGTTGTTCTCTATTAAAAGATACAATCCTGT	67351
67352	GATCGATAACTACCCCGAATAACAGGGGACGAGAAT	TCTGTAATGTATTCATTTAATGTAATCATAATTTTTTC	67425
67426	GATCGATAACTACCCCGAATAACAGGGGACGAGAAT	TAGACCATTTACCTCATTATATTTATAGTCTTTATTA	67498
67499	GATCGATAACTACCCCGAATAACAGGGGACGAGAAT	TTTTCTTTAACTGTTTTTACTGCCCATTTAATAGT	67569
67570	GATCGATAACTACCCCGAATAACAGGGGACGAGAAT	ATAAACCCGTTCAATTCGTTATCTTTAAATTCTTG	67640
67641	GATCGATAACTACCCCGAATAACAGGGGACGAGAAT	ACAACTTCGTCATCTTTCATCATTTCTCTTACATCA	67712
67713	GATCGATAACTACCCCGAATAACAGGGGACGAGAAT	ATATTTCTTCCATGAATAACACCCTCCTTTTTTCTA	67784
67785	GATCGATAACTACCCCGAATAACAGGGGACGAGAAT	AAGTTAACGGCATTACCTAATAAAAATATTTTAGG	67855
67856	GATCGATAACTACCCCGAATAACAGGGGACGAGAAC	TCATCTTTCATGTCACTGATTAATTCATTTGTA	67924
67925	GATCGATAACTACCCCGAATAACAGGGGACGAGAAC	GGTAATAGTTGCTCAATAGGTAATAAAACGTCGGT	67995
67996	GATCGATAACTATCCCGAATAACAGGGGACAGAGTG		68031

76758	ATTCGATAACTACCCCGTAGAAGAGGGGACGAGAACT	CTTCTAAGACGCGATATGATTCTAATTGGTCTTCA	76829
76830	TTCGATAACTACCCCCGTAGAAGAGGGGACGAGAACT	GATATACTCCTTTACCATGTATTAATTCTGGACCACT	76903
76904	ATTCGATAACTACCCCGTAGAAGAGGGGACGAGAACT	CATATTCGATCGTGTATATCAAAACTTTATGC	76972
76973	ATTCGATAACTACCCCGTAGAAGAGGGGACGAGGATC		77009

1549038	GATTAGTATTCATTTAAAATTGCAGAGTACTAAAAC	CTTCCGAATCCATTTCAGCGCAATAAACA	1549102
1549103	GTTTCATTTATACCTAAAATTACAGAGTACTAAAAC	GATTGATAGTTTGCGATTCTTGCAGTCATT	1549168
1549169	GTTTCATTTATACCTAAAATTACAGAGTACTAAAAC	ACTCACTTGTAAATTCCTCCACTTGCTCTA	1549234
1549235	GTTTCATTTATACCTAAAATTACAGAGTACTAAAAC	TCGTCATCAATCCATCGCCATATGCTTCTT	1549300
1549301	GTTTCATTTATACCTAAAATTACAGAGTACTAAAAC	TGACAACCACGCTTTTAGCTTGCATATCAA	1549366
1549367	GTTTCATTTATACCTAAAATTACAGAGTACTAAAAC	ATATTCTATTCAATTCGTTCTGCAACTTGT	1549432
1549433	GTTTCATTTATACCTAAAATTACAGAGTACTAAAAC	TTACATAATCGTTTATGATTATTTCATGTT	1549498
1549499	GTTTCATTTATACCTAAAATTACAGAGTACTAAAAC	CTGGAATAACCACAAAGCCAGAGTCAGTTT	1549564
1549565	GTTTCATTTATACCTAAAATTACAGAGTACTAAAAC	CCATGATGTCTAAAAACACCGATTTACCAT	1549630
1549631	GTTTCATTTATACCTAAAATTACAGAGTACTAAAAC	ACGTTAGATTTGCAGGTGTTAAGCACGGCT	1549696
1549697	GTTTCATTTATACCTAAAATTACAGAGTACTAAAAC	TGTATGGGGTGCGGTCGACTCTACTATCA	1549761
1549762	GTTTCATTTATACCTAAAATTACAGAGTACTAAAAC	GTAAATAATTCCGTTTGGACTCGTTACGTT	1549827
1549828	GTTTCATTTATACCTAAAATTACAGAGTACTAAAAC	CCATCGCTTCTTTGCGTGAATTTGAAATAT	1549893
1549894	GTTTCATTTATACCTAAAATTACAGAGTACTAAAAC	ATGTTTTTTCATTAAAGCTACCAGTAATTC	1549959
1549960	GTTTCATTTATACCTAAAATTACAGAGTACTAAAAC	AATTCGCCTTTATATTCAGGTTTCTTTTTT	1550025
1550026	GTTTCATTTATACCTAAAATTACAGAGTACTAAAAC		1550061

Figure S1. Sequence structure of CRISPR loci discovered in coagulase-negative *Staphylococcus* strains from human origin described in this work.

Strain: *Staphylococcus simulans* FDAARGOS_124
Genbank assess: NZ_CP014016
CRISPR Location: 1194340-1195029 and 2570468-2571030

1194340	TGGTCAATTCATTCTAAAAGATCACAATTCTAAAAC	CTTTCTAGTGCTAACTTGCGTGTATTATC	1194404
1194405	GTTTTACTTCATTCTAAAAGATCACAATTCTAAAAC	CAAAAGAACGTATTAATGGAAAACAATTA	1194469
1194470	GTTTTACTTCATTCTAAAAGATCACAATTCTAAAAC	TAGTGACAACAAAGCGAAAATCGAGATTA	1194534
1194535	GTTTTACTTCATTCTAAAAGATCACAATTCTAAAAC	AAGTGGGCGACAAGTATTTGAAAATTGCA	1194599
1194600	GTTTTACTTCATTCTAAAAGATCACAATTCTAAAAC	AAGTGGGCGACAAGTATTTAAAAATTGCA	1194664
1194665	GTTTTACTTCATTCTAAAAGATCACAATTCTAAAAC	AGCGGTAAACCCGTATTGATTAAAGCAAAG	1194730
1194731	GTTTTACTTCATTCTAAAAGATCACAATTCTAAAAC	GATGCATCAAATGTTTTTACGCCCAATTCA	1194796
1194797	GTTTTACTTCATTCTAAAAGATCACAATTCTAAAAC	TGTGACTTACCTAAATCTAACATTTGATAT	1194862
1194863	GTTTTACTTCATTCTAAAAAATCACAATTCTAAAAC	ATTACGGAGGATGTCATCGGCTTTCTAACA	1194928
1194929	GTTTTACTTCATTCTAAAAGATCACAATTCTAAAAC	AAGTTTTGTGTGTTTCATGATACAGGAAA	1194993
1194994	GTTTTACTTCATTCTAAAAGATCACAATTCTAAAAC		1195029
2570468	GTTTTAGTACTCTGTAATTTTAGGTATGAGTGATTC	TGTTTTAAATGCTTCTAAAGAAAGTGCTTT	2570533
2570534	GTTTTAGTACTCTGTAATTTTAGGTATGAGTGATTC	ATGAACTCAATATCAGTGATAAGCGATTT	2570598
2570599	GTTTTAGTACTCTGTAATTTTAGGTATGAGTGATTC	CAAGGTGAGCGTGTTTATCCGAACAAAAAG	2570664
2570665	GTTTTAGTACTCTGTAATTTTAGGTATGAGTGATTC	TTGAACATATATGAAAGCTCACTGTAACCC	2570730
2570731	GTTTTAGTACTCTGTAATTTTAGGTATGAGTGATTC	TTGTAGCATAACAACAAACTGTGCTCCTAA	2570796
2570797	GTTTTAGTACTCTGTAATTTTAGGTATAAGTGATTC	AAAGCAGTATGAATGCAACAACAAAATCCT	2570862
2570863	GTTTTAGTACTCTGTAATTTTAGGTATAAGTGATTC	AAAGTAATATCAATACAAATCGTATGGCAA	2570928
2570929	GTTTTAGTACTCTGTAATTTTAGGTATAAGTGATTC	ATTAAGTTAGGCTTTGCAGATGAGAAATCA	2570994
2570995	GTTTTAGTACTCTGTAATTTTAAGTATGTGTAATTA		2571030

Strain: *Staphylococcus warneri* 691_SWAR
Genbank assess: NZ_JUWX00000000
CRISPR Location: scaffold NZ_JUWX01000029.1, 33030-33255

33030	GTTTTAGTACTCTGTAATTTTAGGTATGA	GTGAAACATATCAAATGCTTAACTTAGGTAAATATC	33094
33095	GTTTTAGTACTCTGTAATTTTAGGTATGA	GTGAAACTCCTGCATACGAAAATACTTCAATCAGTGC	33160
33161	GTTTTAGTACTCTGTAATTTTAGGTATGA	GTGAAACGCTTTTTGAAAAATACAACTGAAGCTTTTA	33226
33227	GTTTTAGTACTCTGTAATTTTAGGTATGA		33255

Strain: *Staphylococcus aureus* 08BA02176
Genbank assess: CP003808
CRISPR Location: 55513-56620, 65343-65594

55513	GATCGATAACTACCCCGAATAACAGGGGACGAGAAT	TCTATAAGTTCATTAATTCCGATACCTAGATTATCT	55584
55585	GATCGATAACTACCCCGAATAACAGGGGACGAGAAT	TTTTTTCCACCCTTTCAGATCATCTATGATCTTG	55654
55655	GATCGATAACTACCCCGAATAACAGGGGACGAGAAT	AATTTTCTAATTCTATAAGTTCATTAATTCCGAT	55724
55725	GATCGATAACTACCCCGAATAACAGGGGACGAGAAT	TATACTATTTACATAATTTTTTATGTGTCTGTCTAC	55796
55797	GATCGATAACTACCCCGAATAACAGGGGACGAGAAT	TAATAGTGTTGTTCTCTATTAAAAGATACAATCCTGT	55869
55870	GATCGATAACTACCCCGAATAACAGGGGACGAGAAT	TAGAATGTTATTATCTAAGTGGTCGATGTATTCC	55939
55940	GATCGATAACTACCCCGAATAACAGGGGACGAGAAT	TCATACTAGCACCCCACTCTCTACTGAACAAGTATCA	56012
56013	GATCGATAACTACCCCGAATAACAGGGGACGAGAAT	CTTAAAATCTAATTGCATTGTTATCAATTCCTTTA	56083
56084	GATCGATAACTACCCCGAATAACAGGGGACGAGAAT	TCTGTAATGTATTCATTTAATGTAATCATAATTTTTTC	56157
56158	GATCGATAACTACCCCGAATAACAGGGGACGAGAAT	TAGACCATTTACCTCATTATATTTATAGTCTTTATTA	56230
56231	GATCGATAACTACCCCGAATAACAGGGGACGAGAAT	TTTTCTTTAACTGTTTTTACTGCCCATTTAATAGT	56301
56302	GATCGATAACTACCCCGAATAACAGGGGACGAGAAT	ATATTTCTTCCATGAATAACACCCTCCTTTTTTCTA	56373
56374	GATCGATAACTACCCCGAATAACAGGGGACGAGAAT	AAGTTAACGGCATTACCTAATAAAAATATTTTAGG	56444
56445	GATCGATAACTACCCCGAATAACAGGGGACGAGAAC	TCATCTTTCATGTCACTGATTAATTCATTTGTA	56513
56514	GATCGATAACTACCCCGAATAACAGGGGACGAGAAC	GGTAATAGTTGCTCAATAGGTAATAAAACGTCGGT	56584
56585	GATCGATAACTATCCCGAATAACAGGGGACAGAGTG		56620

65343	ATTCGATAACTACCCCGTAGAAGAGGGGACGAGAACT	CTTCTAAGACGCGATATGATTCTAATTGGTCTTCA	65414
65415	TTCGATAACTACCCCCGTAGAAGAGGGGACGAGAACT	GATATACTCCTTTACCATGTATTAATTCTGGACCACT	65488
65489	ATTCGATAACTACCCCGTAGAAGAGGGGACGAGAACT	CATATTCGATCGTGTATATCAAAACTTTATGC	65557
65558	ATTCGATAACTACCCCGTAGAAGAGGGGACGAGGATC		65594

Figure S1. Sequence structure of CRISPR loci discovered in coagulase-negative *Staphylococcus* strains from human origin described in this work.
