# Supplementary material for: CRISPR-Cas Systems Features and the Gene-Reservoir Role of Coagulase-Negative Staphylococci
Source: Front Microbiol. 2017 Aug 15;8:1545. doi: 10.3389/fmicb.2017.01545 (PMC5559504; doi:10.3389/fmicb.2017.01545)
Supplement: Supplementary file 5 [file Image_2.PDF]

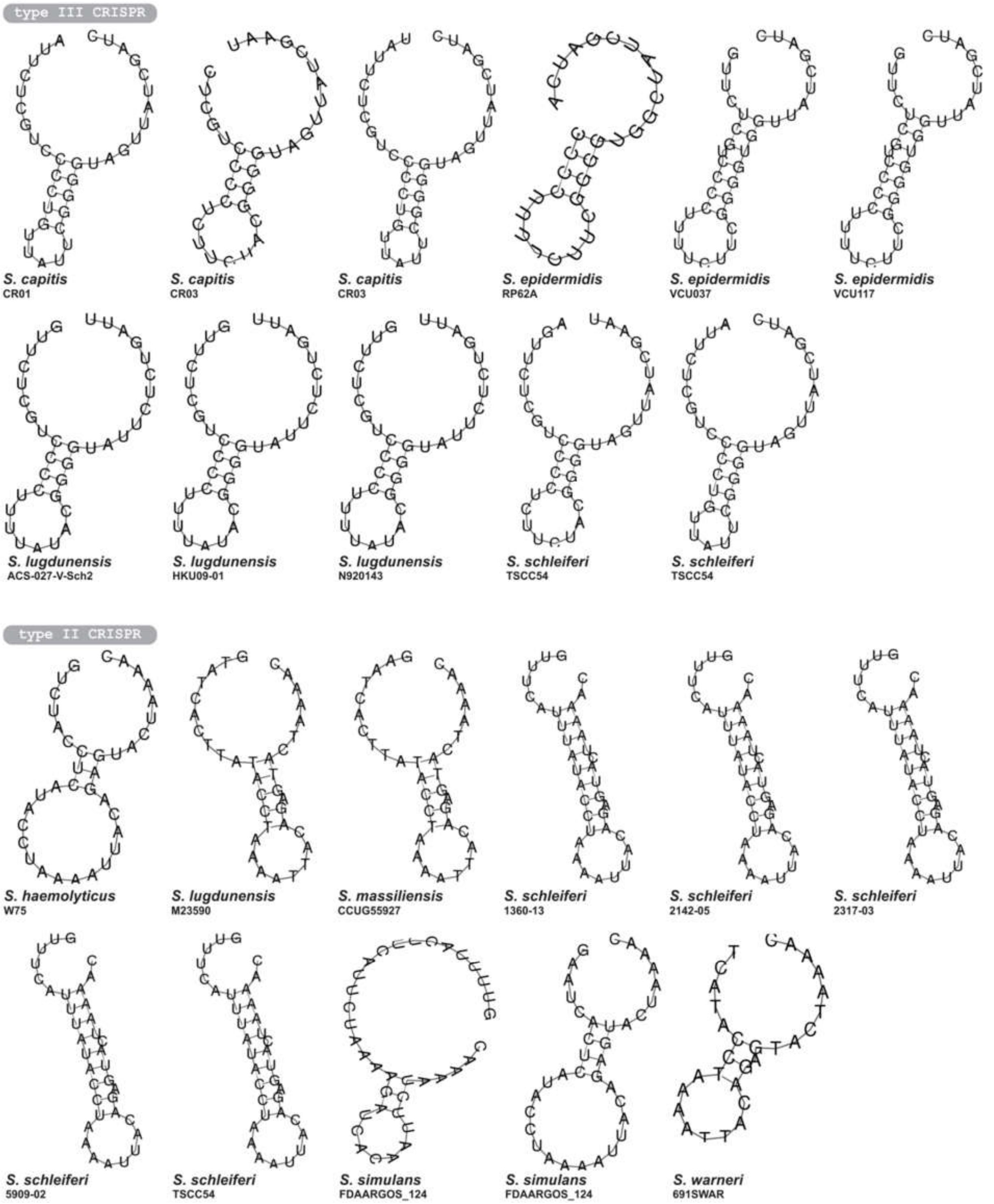

Figure S2. Hairpin secondary structures formed by the direct repeats from each CRISPR found in coagulase negative *Staphylococcus* studied in this work. Structures were predicted by RNAfold. Reference: Gruber AR, Lorenz R, Bernhart SH, Neuböck R, Hofacker IL. (2008). The Vienna RNA Websuite. Nucleic Acids Res. 36 (2): W70-W74.
